# Matrix- and Differentiation Stage-Dependent Variability of Reference Genes: Rethinking Validation Strategies in 3T3-L1 Adipogenic Models

**DOI:** 10.3390/ijms27125268

**Published:** 2026-06-10

**Authors:** Betina Todorova, Zhenya Ivanova, Natalia Grigorova

**Affiliations:** Department of Pharmacology, Animal Physiology, Biochemistry and Chemistry, Faculty of Veterinary Medicine, Trakia University, 6000 Stara Zagora, Bulgaria; betina.todorova@trakia-uni.bg (B.T.); zhenya.ivanova.12@trakia-uni.bg (Z.I.)

**Keywords:** adipogenesis, 3T3-L1 cells, reference gene validation, RT-qPCR normalization, housekeeping genes, extracellular matrix, Matrigel, collagen, gene expression stability, multifactorial experimental design

## Abstract

The present study evaluated the stability of candidate reference genes during adipogenic differentiation of 3T3-L1 cells cultured on different extracellular matrices. The aim was to investigate the effects of matrix composition and differentiation stage on the expression of candidate housekeeping genes and to compare validation strategies in dynamic in vitro models. Eleven candidate reference genes (*18S*, *Actb*, *B2m*, *Gapdh*, *Hmbs*, *Hprt*, *Nono*, *Ppia*, *Rplp0*, *Tbp*, and *Ywhaz*) were analyzed by RT-qPCR in 3T3-L1 cells cultured on TC, collagen, gelatin, and Matrigel at Days 7 and 14 of differentiation. Gene stability was assessed using geNorm, NormFinder, RefFinder, comparative ΔCt, BestKeeper, generalized linear model (GLM), linear mixed model (LMM), and correlation analyses with the adipogenic markers *Pparg* and *Fasn*. The results demonstrated that the expression of most housekeeping genes was influenced by matrix composition, differentiation stage, or their interaction. *Actb* and *18S* exhibited the strongest condition-dependent variability and pronounced matrix sensitivity. *Gapdh* and *Hprt* showed significant correlations with both *Pparg* and *Fasn*, while *Hmbs* correlated with *Fasn*, suggesting that these reference genes may not be fully independent of adipogenic status. *Ppia* demonstrated markedly contrasting rankings across analytical approaches, highlighting limitations of single-method stability assessment. The findings confirm that universal housekeeping genes are unlikely to exist across different matrix conditions and differentiation stages. The results highlight the need for multi-level validation strategies and experimentally validated normalization panels to minimize normalization bias and avoid misleading RT-qPCR expression profiles. Functional validation identified *B2m* and *Rplp0* as the most suitable two-gene normalization panel for the experimental model evaluated, whereas *Tbp* remained a strong complementary reference gene candidate.

## 1. Introduction

Obesity is a chronic multifactorial disease characterized by excessive fat accumulation and an increased risk of metabolic and cardiovascular complications [1]. Despite extensive study, it remains a major global health challenge, underscoring the need for deeper insight into adipose tissue biology and its regulatory mechanisms [2].

To investigate the molecular basis of adipogenesis and lipid metabolism in vitro, the 3T3-L1 cell line has become one of the most widely used and well-characterized models [3,4,5]. Under appropriate induction conditions, these murine preadipocytes undergo a differentiation program involving transcriptional reprogramming, cytoskeletal remodeling, and lipid accumulation, ultimately acquiring the morphological and metabolic features of mature adipocytes. First described by Green and colleagues [3], this model provides a robust and reproducible system for studying the transcriptional and functional dynamics of adipogenesis.

However, the marked biological variability associated with adipogenesis poses a significant challenge to quantitative reverse transcription polymerase chain reaction (RT-qPCR) normalization, particularly in selecting stable reference genes. In such a highly dynamic system, reliance on so-called “classical” housekeeping genes may lead to inaccurate normalization [6,7,8]. As early as 2014, it was demonstrated that during 3T3-L1 differentiation, glyceraldehyde 3-phosphate dehydrogenase (*Gapdh*) is among the least stable reference genes, likely due to its sensitivity to changes in glycolytic activity [7,9]. In addition, other commonly used housekeeping genes, including β-actin (*Actb*), 18S ribosomal RNA (*18S*), hypoxanthine guanine phosphoribosyl transferase (*Hprt*), and beta-2 microglobulin (*B2m*), have been reported to exhibit variable expression depending on the stage of differentiation, metabolic state, and culture conditions [10,11]. *Actb* expression is influenced by cytoskeletal reorganization, whereas ribosomal RNA genes, such as 18S rRNA, display distinct abundance and regulatory patterns compared to mRNA transcripts, further limiting their suitability for normalization in dynamic biological systems. Building upon recent work, including our own studies addressing normalization challenges in 3T3-L1 and related in vitro models, we demonstrated that reference gene stability is highly species-specific: while *Gapdh* may be suitable for rabbit adipose-derived stem cells (ADSc), *Hprt* is the superior choice for equine ADSc. Furthermore, lipid-modulating factors, such as docosahexaenoic acid and palmitic acid, have been shown to significantly destabilize commonly used reference genes in 3T3-L1 cells, necessitating the use of more robust panels, such as peptidylprolyl isomerase A (*Ppia*) and ribosomal protein, large, P0 (*Rplp0*) [12,13]. Commonly used stability assessment tools, including geNorm, NormFinder, BestKeeper, and integrative platforms such as RefFinder, may yield inconsistent results in complex experimental designs. Such discrepancies arise from differences in data input, algorithmic assumptions, and handling of variability, leading to divergent rankings of candidate reference genes [12,13,14,15]. This underscores the need for rigorous context-aware validation strategies that integrate multiple algorithms and statistical approaches to ensure accurate, reproducible, and biologically meaningful RT-qPCR results.

The challenge becomes even more pronounced when, in addition to high metabolic dynamics, the cellular microenvironment is considered. Most in vitro adipogenesis studies are conducted on rigid polystyrene surfaces that poorly recapitulate the biophysical properties of adipose tissue in vivo [16]. Consequently, there is a growing shift toward more physiologically relevant culture systems employing extracellular matrix-derived or biomimetic substrates, including collagen-, gelatin-, and Matrigel-based matrices, in order to better reproduce adipose tissue microenvironmental cues [16,17,18,19,20]. However, the intrinsic effects of these substrates on cellular morphology and metabolic activity remain insufficiently characterized. It is increasingly recognized that substrate-dependent effects are mediated through integrin–focal adhesion kinase (FAK) signaling and mechanotransduction pathways, which modulate cell–matrix interactions, intracellular signaling, and differentiation outcomes [21,22].

Therefore, the present study aims to evaluate the expression stability of eleven commonly used reference genes (*Ppia*, *Gapdh*, *B2m*, *Rplp0*, hydroxymethylbilane synthase (*Hmbs*), *Hprt*, tyrosine 3-monooxygenase/tryptophan 5-monooxygenase activation protein, zeta polypeptide (*Ywhaz*), *18S*, *Actb*, non-POU-domain-containing, octamer-binding protein (*Nono*), and TATA-box binding protein (*Tbp*)) in 3T3-L1 cells cultured on substrates with distinct biochemical and biophysical properties, including standard polystyrene (TC), Collagen I (Coll), Gelatin peptone (GP), and Matrigel (M). In addition, two target genes (*Pparg* and *Fasn*) were included to assess the impact of reference gene selection on downstream gene expression analyses. Classical stability assessment methods (geNorm, NormFinder, BestKeeper, comparative ΔCt, and RefFinder) were systematically compared with each other as well as with linear mixed models (LMMs) to improve reference gene validation strategies in complex, multifactorial experimental designs.

## 2. Results

### 2.1. Phenotypic Validation of Matrix-Dependent Adipogenic Differentiation in 3T3-L1 Cells

**Figure 1:**
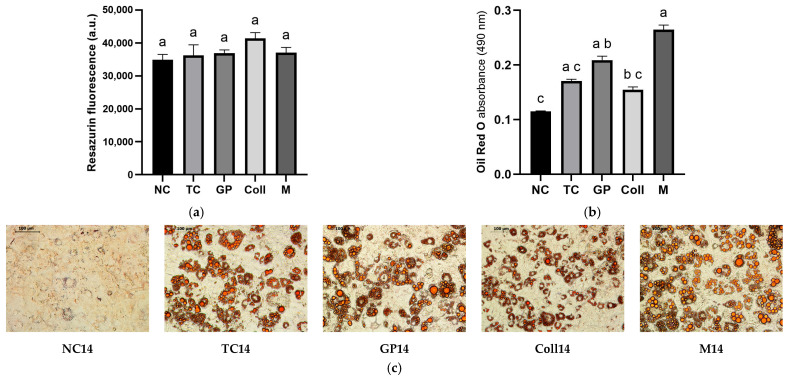
Substrate-dependent metabolic activity and adipogenic differentiation of 3T3-L1 cells. (**a**) Metabolic activity assessed by resazurin assay on day 14 under different substrate conditions, expressed as fluorescence intensity (a.u.; mean ± SEM). (**b**) Quantification of intracellular lipid accumulation by ORO staining on day 14. Lipids were extracted with isopropanol and absorbance measured at 490 nm (mean ± SEM). (**c**) Microscopic images of 3T3-L1 cells, cultured on standard tissue culture plastic under non-differentiated control conditions (NC) and following adipogenic induction on different substrates (TC, GP, Coll and M), were acquired using an inverted microscope (Leica DMi1; 20×; scale bar = 100 µm). Statistical significance was evaluated using the Kruskal–Wallis test (*p* < 0.001) followed by Dunn’s post hoc test; groups denoted by different letters differ significantly at *p* < 0.05. Abbreviations: NC—non-differentiated control; TC—tissue culture plastic; GP—gelatin peptone; Coll—collagen type I; M—Matrigel; ORO—Oil Red O.

To validate the biological relevance of the experimental model, metabolic activity and adipogenic differentiation of cells in all experimental groups were assessed at the end of the study (day 14). Metabolic activity, as determined by the resazurin assay, did not differ significantly. Still, a trend towards increased activity was observed in cells cultured on a collagen coating (Figure 1a).

Adipogenesis was assessed by morphological analysis and quantitative determination of Oil Red O on day 14 (Figure 1b,c). The negative control (NC) demonstrated weak to minimal spontaneous adipogenesis. At the same time, in the treated groups, a clear differentiation was observed, characterized by the formation and accumulation of lipid droplets distinctive of mature adipocytes (Figure 1c).

Quantitative analysis with Oil Red O confirmed successful differentiation and revealed substrate-dependent differences in lipid accumulation. The highest absorption was reported in the group grown on Matrigel, followed by GP and TC (Figure 1b). At the same time, cells cultured on collagen exhibited the lowest lipid accumulation under differentiated conditions.

### 2.2. Classical Stability Analyses of Candidate Reference Genes

The stability of candidate reference genes was initially evaluated using classical algorithms, including NormFinder, geNorm, BestKeeper (descriptive analysis), and the comparative ΔCt method.

All approaches consistently identified *Actb*, *Ppia*, and *18S* as the most unstable genes. In contrast, although general trends were observed regarding the most stable candidates, notable differences in gene ranking emerged across methods. Specifically, geNorm ranked *Rplp0*, *Hmbs*, *B2m*, *Ywhaz*, *Hprt*, and *Tbp* among the most stable genes, whereas NormFinder prioritized *Tbp*, *B2m*, and *Rplp0*. Despite these differences, a consistent overlap was observed, with *B2m* and *Rplp0* being identified as highly stable by both algorithms.

Expression variability-based analyses (comparative ΔCt) further supported these findings, consistently identifying *B2m*, *Rplp0*, *Hmbs*, *Hprt*, *Ywhaz*, and *Tbp* as the least variable genes. Similarly, the integrative RefFinder approach, which combines results from multiple algorithms based on raw Ct values, identified *B2m*, *Rplp0*, and *Hmbs* as the most stable reference genes and ranked *Tbp*, *Hprt*, and *Ywhaz* among the most appropriate candidates.

According to the geNorm pairwise variation analysis, two reference genes were sufficient for normalization, as the V2/3 value (0.090) was below the recommended threshold of 0.15. All subsequent Vn/Vn + 1 values also remained below this threshold, indicating that inclusion of additional reference genes was not required (Supplementary Table S1).

**Figure 2:**
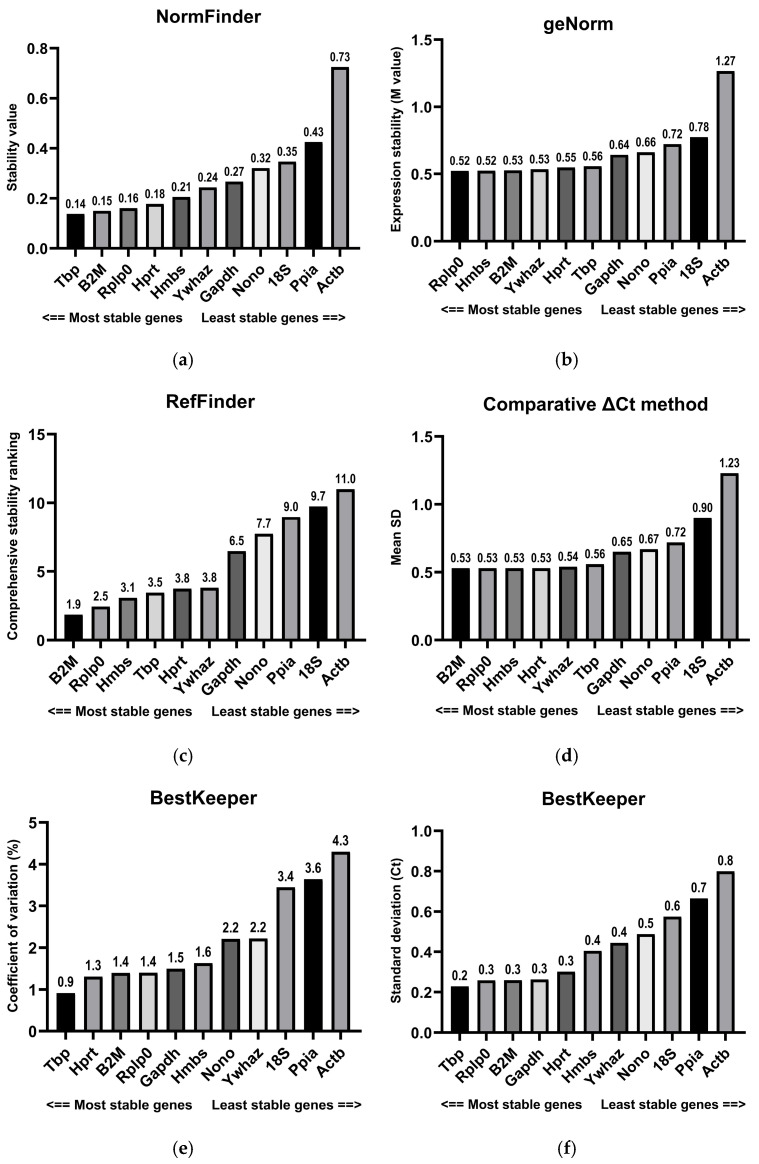
Stability ranking of candidate reference genes using classical algorithms and Ct variability-based analyses. Expression stability of candidate reference genes was evaluated using NormFinder (**a**), geNorm (**b**), RefFinder (**c**), the comparative ΔCt method (**d**), and BestKeeper-based variability analyses, including coefficient of variation (CV, %) (**e**) and standard deviation (SD) (**f**).

To confirm the classical approaches evaluated so far, gene stability was further estimated using BestKeeper analyses of Ct variability (SD and CV). As shown in Figure 2e,f, *Tbp*, *Hprt*, *B2m*, and *Rplp0*, together with *Gapdh*, were identified as genes exhibiting comparatively low variability.

### 2.3. Correlation and Linear Regression Analysis of Candidate Reference Genes Against the BestKeeper Index

However, when gene stability was assessed based on the correlation coefficient with the BestKeeper index, a slightly different pattern emerged. Strong positive correlations were observed for *Ppia*, *Ywhaz*, *B2m*, and *Tbp*, indicating that these genes closely follow the overall expression trend represented by the index. In contrast, *Gapdh*, *Hprt*, and *18S* showed weak correlations, while *Actb* exhibited a negative and non-significant relationship, indicating poor overlap with the global expression profile (Table 1).

**Figure 3:**
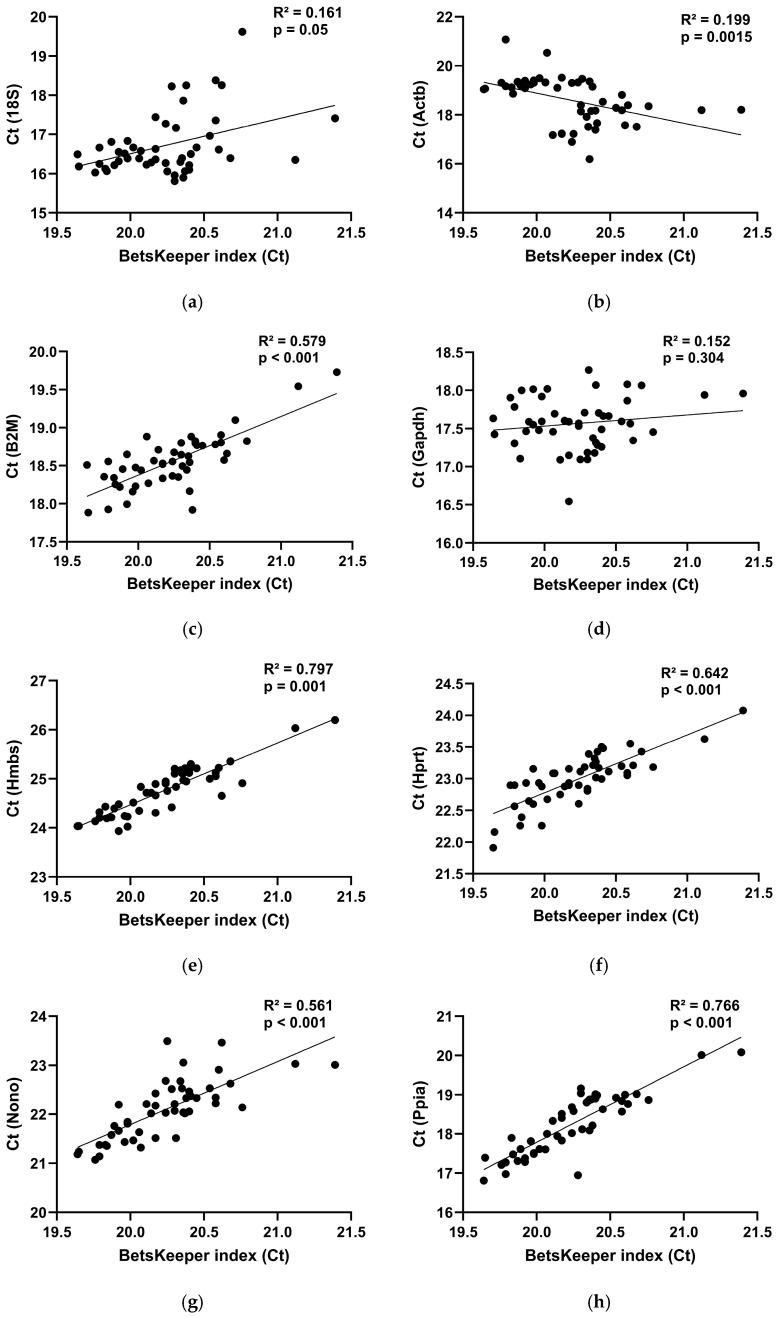

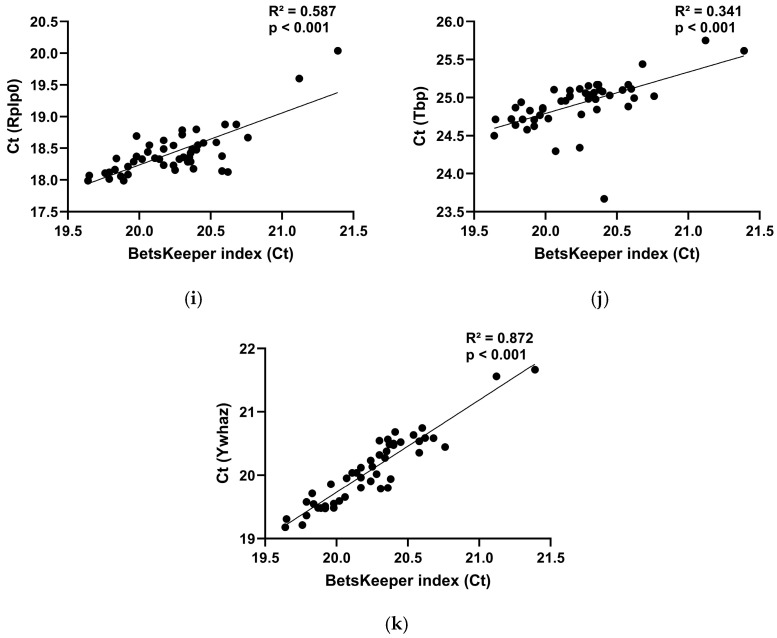
Linear regression analysis of candidate reference genes against the BestKeeper index. Linear regression analysis was performed to assess the relationship between individual gene Ct values and the BestKeeper index (Ct) across all experimental conditions. Each panel represents a separate candidate reference gene: (**a**) *18S*, (**b**) *Actb*, (**c**) *B2m*, (**d**) *Gapdh*, (**e**) *Hmbs*, (**f**) *Hprt*, (**g**) *Nono*, (**h**) *Ppia*, (**i**) *Rplp0*, (**j**) *Tbp*, and (**k**) *Ywhaz*. Scatter plots display individual samples, with the BestKeeper index plotted on the *x*-axis and gene-specific Ct values on the *y*-axis. Solid lines represent linear regression fits. Coefficients of determination (R^2^) and corresponding *p*-values are indicated within each panel.

These observations were further supported by a linear regression analysis of individual gene Ct values against the BestKeeper index (Figure 3). Genes such as *Ywhaz*, *Ppia*, and *Hmbs* demonstrated strong linear relationships, reflected by high coefficients of determination, indicating consistent proportional changes relative to the overall expression pattern. In contrast, *Gapdh*, *18S*, and *Actb* displayed weak or non-significant regression fits, suggesting increased variability and reduced predictability.

### 2.4. Pairwise Spearman Correlation Analysis Among Candidate Reference Genes

Spearman’s correlation analysis was performed to evaluate the relationship between the expression of the target genes (peroxisome proliferator-activated receptor gamma (*Pparg*) and fatty acid synthase (*Fasn*)) and the candidate reference genes across all experimental conditions (Table 2). To account for multiple testing, p-values were additionally adjusted using the Benjamini–Hochberg false discovery rate (FDR) procedure. For *Pparg*, only a limited subset of reference genes showed significant positive correlations after FDR correction. Moderate correlations were observed with *Gapdh* and *Hprt* (FDR-adjusted *p* = 0.033). Although *Hmbs* and *Tbp* showed nominal significance in the unadjusted analysis, these associations were no longer significant after FDR correction. No significant correlation was found between *Pparg* expression and several commonly used reference genes, including *18S*, *Actb*, *B2m*, *Nono*, *Ppia*, *Rplp0*, and *Ywhaz*, indicating limited coherence in their expression patterns.

A similar trend was observed for *Fasn* (Table 2). Significant positive correlations after FDR correction were identified with *Gapdh* (FDR-adjusted *p* = 0.011), *Hprt* (FDR-adjusted *p* = 0.033), and *Hmbs* (FDR-adjusted *p* = 0.040). All other genes, including *Tbp*, *18S*, *Actb*, *B2m*, *Nono*, *Ppia*, *Rplp0*, and *Ywhaz*, showed no significant correlation with *Fasn* expression after multiple-testing correction.

### 2.5. LMM Analysis of Reference Gene Stability

An LMM was applied to evaluate the effects of gene identity, matrix type, passage, and culture duration on Ct values across the full dataset. The analysis revealed no significant effect of passage (F = 0.17, *p* = 0.687). In contrast, highly significant main effects were observed for gene (F = 4812.23, *p* < 0.001), matrix (F = 9.48, *p* < 0.001), and day (F = 28.72, *p* < 0.001), demonstrating that Ct values differed substantially among candidate reference genes and were influenced by both extracellular matrix conditions and differentiation stage (Table 3).

Significant interaction effects were detected for gene × matrix (F = 3.96, *p* < 0.001), gene × day (F = 83.13, *p* < 0.001), and gene × matrix × day (F = 6.39, *p* < 0.001). In contrast, the overall matrix × day interaction was not statistically significant (F = 1.43, *p* = 0.248), indicating that the effects of matrix composition and differentiation stage on Ct values were strongly gene-dependent (Table 3).

Estimates of Fixed Effects are presented in Supplementary Table S2. Pronounced condition-dependent variability for *18S*, particularly under Matrigel conditions, was observed (*p* < 0.001). In addition, *18S* exhibited significant differentiation stage-dependent variability at day 7 and the strongest three-way interaction effects, indicating marked sensitivity to the combined influence of extracellular matrix composition and adipogenic maturation (Supplementary Table S2). Similar matrix-associated effects were evaluated for *Actb*, with significant Ct shifts under collagen, gelatin peptone, and Matrigel. *Actb* also showed the strongest differentiation-associated effect overall (Estimate = 2.03, *p* < 0.001) together with significant three-way interactions.

*Gapdh* likewise demonstrated significant condition-dependent variability, including Matrigel-associated changes (Estimate = −0.50, *p* = 0.036), a significant day-dependent effect (Estimate = 0.48, *p* = 0.043), and multiple significant three-way interactions involving matrix composition and differentiation stage. *Nono* displayed selective variability under Matrigel influence at day 7 (Estimate = 1.01, *p* = 0.003).

*Tbp*, *Rplp0*, and *Hprt* showed no significant matrix-dependent and three-way interaction effects, but indicated modest differentiation stage-related variability at day 7.

Limited condition-dependent variability in the LMM interaction analysis was established for *B2m*, *Hmbs*, and *Ppia*, as none of them exhibited significant matrix, day, or three-way interaction variability.

### 2.6. Gene-Specific Effects Revealed by GLM

To further refine the gene-specific effects identified by the LMM, a two-way analysis of variance (GLM) was performed separately for each gene (gene-by-gene approach), with passage, matrix, and day as fixed factors, including the matrix × day interaction. Passage was included in order to account for potential variability between the two closely related cell passages analyzed in parallel. This approach allowed direct evaluation of each factor’s contribution to Ct variability and facilitated a more precise assessment of reference gene stability.

The GLM analysis demonstrated that passage-associated variability was negligible for nearly all candidate reference genes, with no significant passage effect detected for *18S*, *Actb*, *B2m*, *Gapdh*, *Hmbs*, *Hprt*, *Ppia*, *Rplp0*, *Tbp*, or *Ywhaz* (Table 4). *Nono* was the only gene exhibiting a modest but significant passage-dependent effect (*p* = 0.015, partial η^2^ = 0.141). The stage of adipocyte maturation (factor: Day) was a major source of variation for nearly all genes, with particularly strong effects observed for *Actb*, *Hmbs*, *Ppia*, and *Ywhaz* (Table 4). Smaller yet significant effects were observed for *Gapdh* and *Tbp*, suggesting comparatively greater stability over time. Only *18S* did not exhibit a significant effect of the Day factor, indicating relative stability throughout adipogenic differentiation.

Significant substrate-dependent variability was detected in several candidate reference genes, with moderate matrix-associated effects for *18S*, *Rplp0*, *Tbp*, *Ppia*, and *Ywhaz*, and stronger effects for *Actb*, and *Hmbs*. Relative stability across different matrices was established for *B2m*, *Gapdh*, *Hprt*, and *Nono*.

The matrix × day interactions were pronounced for *Gapdh*, *Nono*, *Rplp0*, *B2m*, *Actb* and *Hprt*, and the strongest interaction was observed for *18S*. Insignificant variability was detected for *Hmbs*, *Ppia*, *Tbp*, and *Ywhaz*.

### 2.7. Functional Validation of the Proposed Normalization Panel

The combined stability analyses identified *Actb* and *18S* as the least suitable reference genes, whereas *Tbp*, *Rplp0*, and *B2m* consistently ranked among the most stable candidates. *Nono* exhibited passage-dependent variability, while *Ppia* was included in the functional validation based on its favorable performance in selected analyses.

**Figure 4:**
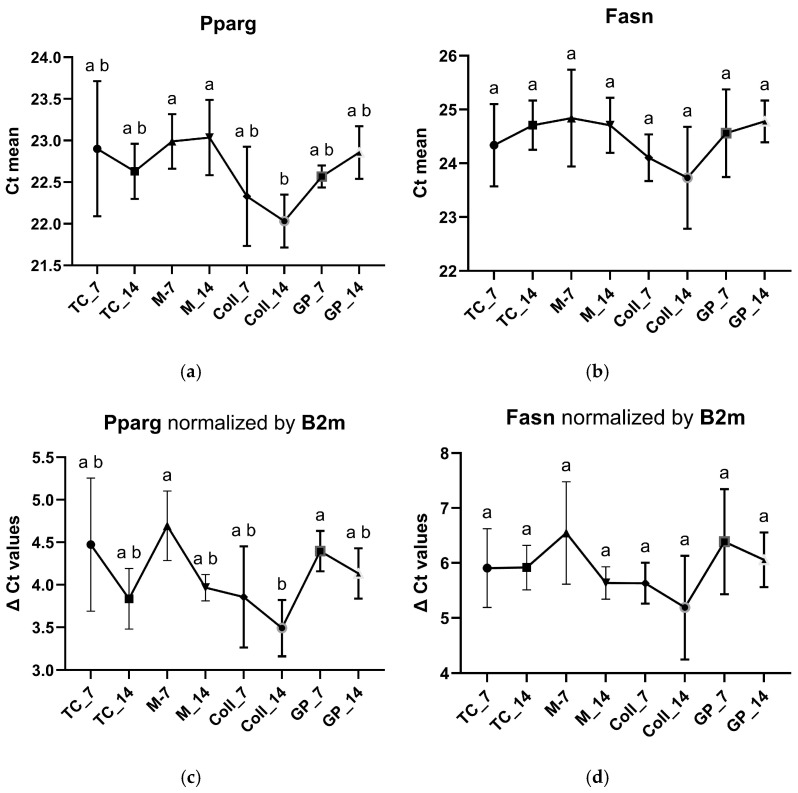

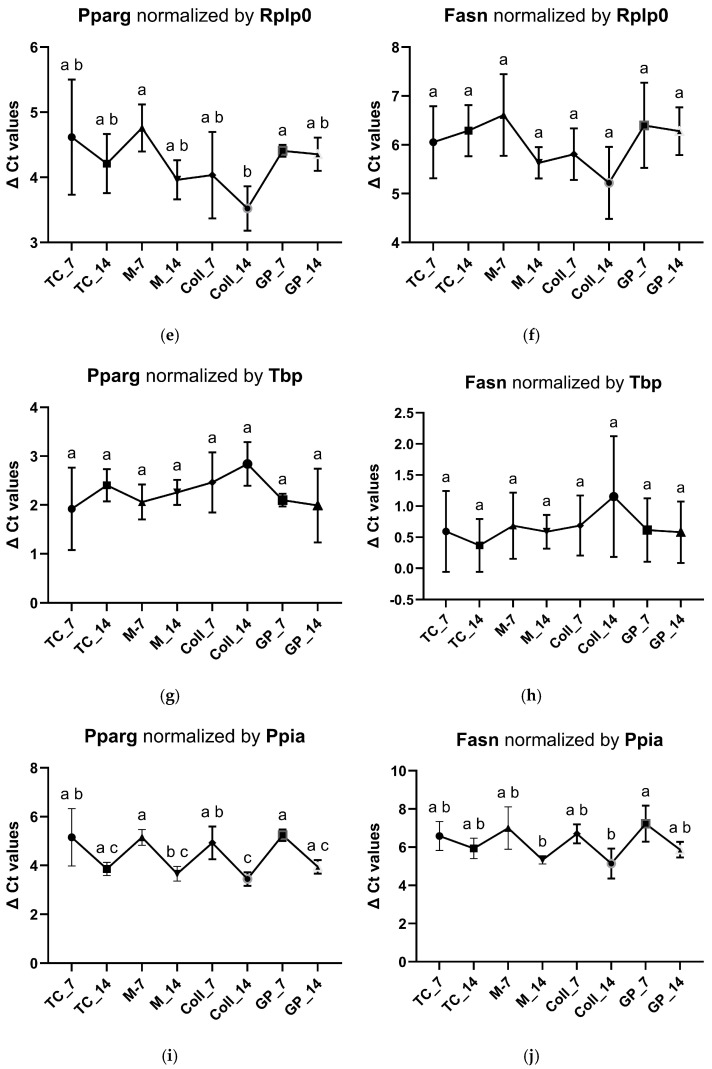
Functional validation of candidate reference genes using adipogenic marker expression profiles. Raw Ct values of *Pparg* (**a**) and *Fasn* (**b**) are shown together with corresponding ΔCt values obtained after normalization using *B2m* (**c**,**d**), *Rplp0* (**e**,**f**), *Tbp* (**g**,**h**), and *Ppia* (**i**,**j**). Data are presented as mean ± SD (*n* = 6). Different letters indicate statistical significance among groups according to Dunn’s multiple-comparison test following Kruskal–Wallis analysis (*p* < 0.05).

To evaluate the practical impact of reference gene selection, the adipogenic markers *Pparg* and *Fasn* were normalized separately using each candidate reference gene. Raw Ct values of both target genes were included for comparison. As shown in Figure 4, normalization using *B2m* and *Rplp0* produced expression profiles that most closely reflected the trends observed in the raw Ct data. In contrast, normalization with *Tbp* moderately altered the relative expression patterns across experimental groups despite its high ranking in several stability analyses. The greatest deviation from the raw Ct tendency was observed following normalization with *Ppia*, which introduced additional differences among groups and modified the overall expression framework of both target genes.

To further evaluate the candidate normalization panel, a repeated BestKeeper analysis was performed using only the four reference genes selected from the integrated stability assessment (*Tbp*, *B2m*, *Rplp0*, and *Ppia*). Among these genes, *B2m* and *Rplp0* exhibited the lowest expression variability, with standard deviations of 0.26 CP and coefficients of variation of 1.40% and 1.41%, respectively (Table 5). *Tbp* showed the lowest coefficient of variation (0.92%), whereas *Ppia* displayed substantially higher variability (SD = 0.67 CP; CV = 3.65%).

Linear regression analysis relative to the newly calculated BestKeeper index confirmed strong correlations for all four candidate reference genes (Table 6). The highest correlation was observed for *Ppia*, followed by *Rplp0* and *B2m*. *Tbp* showed the weakest association with the BestKeeper index among the candidate reference genes (r = 0.635, R^2^ = 0.403, *p* = 0.001). The adipogenic marker genes *Pparg* and *Fasn* were not significantly correlated with the BestKeeper index, supporting their independence from the selected normalization panel.

## 3. Discussion

Modern adipocyte culture models increasingly employ physiologically relevant substrates, such as collagen-, Matrigel-, and gelatin-based matrices, to better replicate the in vivo microenvironment [23,24]. Nevertheless, the extent to which different matrix conditions influence housekeeping gene stability during adipogenic differentiation remains insufficiently understood. The findings herein demonstrate that even morphologically similar differentiated adipocytes retain sensitivity to mechanical and biochemical signals originating from the extracellular matrix. The observed matrix-associated effects in several candidate reference genes suggest that matrix composition may directly influence basal transcriptional activity and consequently affect the expression stability of housekeeping genes. The present study provides a comprehensive evaluation of reference gene stability in a multifactorial 3T3-L1 adipogenesis model incorporating both different stages of differentiation and variations in extracellular matrix composition.

Classical stability algorithms, including geNorm, NormFinder, comparative ΔCt, and the descriptive BestKeeper analysis based on all candidate reference genes [25,26,27], produced relatively consistent results for several candidate genes, with *B2m*, *Rplp0*, *Hmbs*, *Tbp*, and *Hprt* being ranked among the most stable genes. However, these approaches primarily rely on overall expression variability and co-regulation patterns between genes without directly accounting for the complexity of multifactorial experimental designs and interaction-associated variability. Genes exhibiting substantial context-dependent variability may still be classified as stable reference controls when assessed exclusively by variance- or correlation-based approaches. Similar limitations of classical normalization algorithms have been reported in studies involving complex biological systems and multifactorial experimental designs [28,29].

An analogous observation emerged from the BestKeeper correlation and regression analyses. Several candidate reference genes, including *Ppia*, *Ywhaz*, *Tbp*, *B2m*, *Rplp0*, and *Hmbs* showed strong agreement with the BestKeeper index, as evident by high correlation coefficients and linear regression fits. However, because the BestKeeper index is derived from the candidate reference genes themselves, strong agreement primarily reflects consistency with the dominant expression pattern of the analyzed gene set rather than condition-independent expression stability. Genes that closely follow the BestKeeper index may still exhibit significant biological responses to experimental factors. These findings further suggest that correlation- and variance-based approaches alone may not be sufficient to identify the most appropriate reference genes in multifactorial experimental models and highlight the need for complementary model-based analyses.

The application of the GLM and LMM enabled the evaluation of the effects of cell passage, matrix composition, differentiation stage, and their interaction on the expression of each analyzed gene. These analyses demonstrated that the stage of adipocyte maturation (Factor “Day”) represented a major source of variability for most genes, whereas matrix-dependent effects differ substantially among housekeeping genes. *Actb* consistently exhibited pronounced condition-dependent variability across statistical approaches, while *18S* showed marked matrix-associated and interaction-related effects. *Nono* was the only gene affected by cell passage, indicating reduced suitability for studies involving multiple passages. In contrast, *B2m*, *Tbp*, and *Rplp0* demonstrated comparatively stable expression profiles. Although individual rankings varied depending on the method applied, these genes showed the greatest overall agreement between classical stability algorithms and model-based analyses.

One of the most illustrative examples of discrepancy between analytical approaches was *Ppia*. Although this gene demonstrated very strong agreement with the BestKeeper index and robust regression characteristics, it was ranked among the least stable candidates by geNorm, NormFinder, RefFinder, comparative ΔCt analysis, and the descriptive BestKeeper statistics (CV and SD). However, strong agreement with the BestKeeper index should not be equated with true expression stability. Because the index is calculated from the candidate reference genes themselves, it primarily reflects the dominant expression pattern within the analyzed gene set. Furthermore, the GLM analysis revealed pronounced differentiation stage dependency together with a moderate matrix effect. In contrast, the LMM analysis identified only limited condition-dependent variability, as no significant matrix, day, or three-way interaction effects were detected. These contrasting results demonstrate that different validation methods capture distinct aspects of expression variability and may therefore lead to conflicting conclusions regarding reference gene suitability.

An important finding of our study was the estimated potential correlation between the expression of several candidate reference genes, including *Gapdh*, *Hmbs*, and *Hprt*, and key adipogenic markers such as *Pparg* and *Fasn*. These genes also demonstrated significant day- and interaction-associated variability, suggesting that their expression may reflect, to some extent, an adipogenic transcriptional trend. This observation breaches one of the fundamental requirements for a reference gene, namely independence from the biological process under investigation. Nevertheless, the possible co-regulation is often underestimated during housekeeping gene validation and is rarely considered in complex multifactorial experimental settings [30,31].

*Actb* was outlined as the most condition-dependent gene in the present study. Pronounced matrix-, differentiation stage-, and interaction-associated effects were consistently identified by both GLM and LMM analyses, together with weak regression performance and negative correlation with the BestKeeper index. These findings are biologically plausible considering the central role of β-actin in cytoskeletal organization, cell adhesion, mechanotransduction, and morphological remodeling during adipogenesis. It is well established that culturing cells on physiologically relevant matrices can substantially affect actin dynamics and cellular mechanosensitivity, which likely explains the high sensitivity of *Actb* to matrix conditions observed in the present model. Such observations regarding the limited suitability of *Actb* as a universal housekeeping gene in models involving cellular differentiation, ECM signaling, and structural remodeling have been reported previously [32,33].

Similarly, *18S* exhibited pronounced matrix-associated variability and strong interaction effects despite the absence of a significant day effect in the GLM analysis. The LMM further revealed strong gene × matrix and gene × day interactions, demonstrating that apparent stability in single-factor analyses may mask substantial context-dependent variability.

Based on the combined evidence obtained from classical stability algorithms and GLM and LMM analyses, and after excluding genes showing potential associations with adipogenic target pattern, *B2m*, *Tbp*, and *Rplp0* emerged as the most suitable candidates for normalization in the present model. Although the relative rankings varied depending on the analytical method applied, these genes consistently demonstrated favorable stability characteristics and were not associated with *Pparg* or *Fasn* expression.

The subsequent functional validation further refined candidate selection. According to the geNorm pairwise variation analysis, the use of two reference genes was sufficient for reliable normalization, indicating that inclusion of additional genes was not required. Comparison of normalized *Pparg* and *Fasn* expression profiles revealed that *B2m* and *Rplp0* most closely preserved the trends observed in the raw Ct data, whereas normalization using *Tbp* moderately altered the expression patterns despite its consistently high stability ranking. This observation was further supported by the repeated BestKeeper analysis, in which *Tbp* exhibited the lowest correlation with the recalculated BestKeeper index among the selected candidate reference genes. Together, these findings suggest that *B2m* and *Rplp0* represent the most suitable two-gene normalization panel for the experimental model investigated.

*Ppia* was retained for functional validation because it represented a particularly informative case of conflicting stability assessments across analytical methods. However, the ΔCt model of *Pparg* and *Fasn* gene expression following normalization with *Ppia* exhibited the greatest deviation from the original target gene profiles.

The present study demonstrates that different validation approaches assess distinct and non-interchangeable aspects of expression variability. The significant matrix and matrix × day effects identified by GLM and LMM analyses revealed context-dependent variability that could not be reliably detected using classical variance- and correlation-based algorithms. These findings challenge the widespread assumption that once validated, a reference gene can be universally applied across different culture conditions and differentiation stages. Notably, several of the most commonly used housekeeping genes, including *Actb*, *18S*, and *Ppia*, exhibited pronounced sensitivity to experimental conditions, further supporting the growing body of evidence questioning the use of traditional reference genes without prior context-dependent validation [12,31,34,35].

Our findings have direct implications for the normalization of RT-qPCR data in complex cellular models. Selection of reference genes based solely on low overall variability or high co-regulation may mask biologically regulated expression patterns and introduce systematic bias into data interpretation. In this context, our investigation highlights the need for integrated, design-dependent validation strategies combining classical algorithms with statistical models capable of accounting for factor-dependent and interaction-associated effects. Such an approach could substantially improve the reliability of RT-qPCR normalization in ECM-dependent cellular systems while reducing the risk of generating misleading biological interpretations.

A limitation of the present study is that reference gene stability was evaluated only at days 7 and 14 of adipogenic differentiation and therefore does not capture the early transcriptional reprogramming phase of adipogenesis. Consequently, the proposed normalization panel should be interpreted as specific to the analyzed differentiation window under the present ECM-dependent culture conditions. In addition, the use of the immortalized murine 3T3-L1 cell line may not fully reflect the heterogeneity of primary adipocytes or human adipose tissue. Therefore, further studies are required to validate the proposed normalization panel across additional stages of adipogenesis and in primary as well as human adipocyte models.

## 4. Materials and Methods

### 4.1. 3T3-L1 Cell Culture and Pre-Differentiation Protocol

Murine preadipocytes 3T3-L1 (CRL-3242, American Type Culture Collection, Washington, DC, USA) were cultured under standard conditions using high-glucose Dulbecco’s Modified Eagle’s Medium (DMEM-HG, 4.5 g/L glucose) supplemented with 10% fetal bovine serum (FBS) and 1% antibiotic mixture (penicillin G, streptomycin, and amphotericin B), referred to as control medium (CM). Cells were incubated at 37 °C in a humidified atmosphere with 5% CO_2_.

After expansion in two T75 culture flasks (passage 4 and 5), cells were seeded into 24-well plates (Corning, Costar) at an initial density of 2 × 10^4^ cells/mL and were allowed to proliferate until a dense monolayer formed. Upon reaching 100% confluence, the preadipocytes were maintained in CM for 24 h to induce growth arrest and synchronize the cell cycle before differentiation.

### 4.2. Adipogenic Differentiation and Experimental Design

Adipogenic differentiation was initiated by replacing the CM with induction medium (IM), consisting of DMEM supplemented with 10 μg/mL insulin, 0.1 mM 3-isobutyl-1-methylxanthine (IBMX), 1 μM dexamethasone, and 0.05 mM indomethacin (Sigma-Aldrich Chemie GmbH, Merck KGaA, Darmstadt, Germany), for a period of 48 h.

Following the induction stage, the cells were cultured in maintenance medium (MM), a CM supplemented with 10 μg/mL insulin, until the end of the experiment. The experiment lasted 14 days, with samples collected on days 7 and 14 for subsequent analysis.

### 4.3. Surface Coating Protocols

To model various cellular microenvironments and their effects on adipogenesis, cells were cultured on different substrates: standard tissue culture plastic (2D control), gelatin-peptone, type I collagen, and Matrigel.

Type I collagen (rat tail, Corning Inc., Corning, NY, USA) was used at a final surface density of 5 µg/cm^2^. The working solution was prepared by diluting the stock solution in cold PBS to a concentration corresponding to the well surface area (approximately 10 µg/mL for a 24-well plate).

Matrigel (Basement Membrane Matrix, Corning) was diluted in cold DMEM to 1% (*v*/*v*) to produce a thin protein layer. This concentration was chosen to match the total protein load of the collagen coating (5 µg/cm^2^) and minimize variation in adhesive substrate amounts across experimental conditions.

Gelatin-peptone (Gelysate Peptone, Gibco, Thermo Fisher Scientific, Waltham, MA, USA) was prepared as a 0.1% solution in PBS and used under the same application conditions.

Since collagen and Matrigel are thermosensitive, all preparation and application steps were conducted at low temperature using pre-chilled solutions, plates, and pipette tips to prevent premature polymerization. After applying 100 µL of each solution per well, plates were incubated at 37 °C for 90 min to form a stable hydrogel layer. Surfaces were then washed with PBS to remove non-polymerized components.

For each matrix condition and differentiation day, six replicate wells were analyzed, consisting of three wells from passage 4 and three wells from passage 5, cultured and differentiated in parallel.

### 4.4. Fluorometric Assessment of Cell Viability

Cell viability and metabolic activity were assessed using a resazurin-based in vitro toxicology assay (Resazurin sodium salt, Thermo Fisher Scientific, Waltham, MA, USA), performed in accordance with the manufacturer’s protocol. Resazurin is a non-toxic, cell-permeable, non-fluorescent blue dye that is reduced by metabolically active cells to resorufin, a pink and highly fluorescent compound, thereby serving as an indicator of cellular metabolic activity and viability. At the selected experimental time points, 10% of the CM in each well was replaced with the same volume of fresh medium supplemented with 1% (*v*/*v*) resazurin reagent. The cells were then incubated under standard culture conditions (37 °C, 5% CO_2_) for 2 h to allow enzymatic conversion of resazurin by viable cells.

Following incubation, 150 µL of the supernatant from each well was transferred to a 96-well plate for spectrophotometric analysis. Fluorescence intensity was measured using appropriate excitation and emission filters (Ex 560 nm/Em 590 nm) with a Synergy™ LX Multi-Mode Microplate Reader (BioTek Instruments, Inc., Winooski, VT, USA). The obtained fluorescence values were used as an indirect quantitative measure of cell viability, proliferation, and metabolic activity under the respective experimental conditions.

### 4.5. Assessment of Intracellular Lipid Accumulation

Neutral lipid accumulation was assessed using Oil Red O (ORO) staining. Following removal of the culture medium, the cells were washed with PBS, fixed with 10% neutral formalin, and treated with 60% isopropanol.

Subsequently, the cells were incubated with freshly prepared Oil Red O working solution for 15 min in the dark, followed by two washes with PBS. Intracellular lipid droplets were visualized using an inverted Leica DMi1 microscope (Leica Microsystems, Wetzlar, Germany) equipped with a 5-megapixel camera.

For quantitative analysis, the bound dye was extracted using 100% isopropanol, and the absorbance of the resulting solution was measured at 490 nm using a Synergy™ LX Multi-Mode Microplate Reader (BioTek Instruments, Inc., Winooski, VT, USA).

### 4.6. RNA Isolation and cDNA Synthesis

Total RNA was isolated using QIAzol Lysis Reagent and the RNeasy^®^ Lipid Tissue Mini Kit (QIAGEN, Hilden, Germany) according to the manufacturer’s instructions. The isolation protocol included column-based purification to minimize contaminant carryover. RNA concentration and purity were determined spectrophotometrically by measuring absorbance at 260 and 280 nm, using Take-3 Microvolume plate of Synergy LX Multi-Mode Microplate Reader (BioTek Instruments, Inc., Santa Clara, CA, USA). The sample OD ratios varied between 1.93 and 2.09. After normalization of RNA concentration across all samples, reverse transcription was performed using the RevertAid First Strand cDNA Synthesis Kit (Thermo Fisher Scientific, Waltham, MA, USA). The obtained cDNA was stored at −20 °C until use and was diluted 1:50 with nuclease-free water before RT-qPCR analysis.

To assess potential genomic DNA contamination, pooled no-reverse-transcription (no-RT) controls were additionally included and showed no detectable or only negligible late amplification (Supplementary Figure S1). No-template controls (NTCs) were included in all RT-qPCR runs. Amplification specificity for each primer pair was confirmed by melting curve analysis and gel electrophoresis, demonstrating single specific amplification products (Supplementary Figure S1 (RT+)).

### 4.7. Quantitative Real-Time PCR (RT-qPCR) Analysis

RT-qPCR analysis was performed using the SYBR Green-based detection method with KAPA SYBR^®^ Fast qPCR Master Mix (Kapa Biosystems, Wilmington, MA, USA) on a PikoReal Real-Time PCR System (Thermo Fisher Scientific, Waltham, MA, USA). All reactions were performed in duplicate according to the manufacturer’s protocols. The expression levels of two key adipogenic target genes—*Pparg* and *Fasn*—were analyzed in parallel with a panel of candidate reference genes. The obtained raw Ct data is presented in Supplementary Table S3.

The specificity of the amplification was rigorously confirmed by post-cycling melt curve analysis. PCR reaction efficiency (*E*) was calculated using standard curves (Supplementary Figure S2), with values ranging from 97% to 106%, ensuring highly reliable quantification. Detailed primer sequences are provided in Table 7.

### 4.8. Reference Gene Selection and Bioinformatic Analysis

The expression stability of candidate reference genes was evaluated using four distinct algorithms: NormFinder, geNorm, BestKeeper, and the comparative ΔCt method. To obtain a comprehensive ranking, the RefFinder tool was utilized to integrate the outputs of these individual algorithms, generating a final overall stability index. This multi-algorithm consensus approach ensured a robust and reliable selection of internal controls, specifically tailored to the requirements of the multifactorial experimental design. For each candidate reference gene, the dataset consisted of 4 matrix conditions (TC, M, Coll, GP) × 2 differentiation days (day 7 and day 14) × 6 replicate wells (three wells from passage 4 and three wells from passage 5), resulting in *n* = 48 Ct measurements per gene.

### 4.9. Statistical Analysis and Data Modeling

Statistical analysis was performed using GraphPad Prism 11.0.1 (GraphPad Software LLC, Boston, MA, USA) and IBM SPSS Statistics (version 26). The LMM was fitted with Ct values as the dependent variable. Gene, matrix condition, differentiation day, passage, and the interactions among gene, matrix condition, and differentiation day were included as fixed effects. SampleID (representing an individual well-derived cDNA sample) was specified as the subject variable, with a random intercept included to account for the correlation among multiple gene measurements obtained from the same sample. Gene-specific GLM analyses were performed separately for each candidate reference gene. Ct values were analyzed using matrix condition, differentiation day, passage, and the matrix × day interaction as fixed effects. Additionally, correlation analysis was performed to assess relationships between the expression of reference and target genes, as well as among different stability evaluation approaches.

Classical statistical tests included one-way ANOVA with Tukey’s post hoc test for normally distributed data or the Kruskal–Wallis test with Dunn’s post hoc analysis for non-normally distributed data. Prior to statistical analysis, data distributions were assessed for normality using the Shapiro–Wilk test. The level of statistical significance was accepted at *p* < 0.05.

## 5. Conclusions

The present study demonstrates that universal housekeeping genes are unlikely to exist in dynamic models of cellular differentiation and ECM-mediated regulation. The obtained data strongly support the need for integrated validation strategies combining classical stability algorithms with statistical models such as GLMs and LMMs capable of accounting for factor-dependent and interaction-associated effects. Furthermore, the findings emphasize the importance of using normalization panels composed of multiple experimentally validated reference genes rather than relying on one or two traditional housekeeping genes selected solely based on literature reports. Such a strategy is essential for minimizing normalization bias, improving the reliability of RT-qPCR normalization, and preventing misleading gene expression profiles in complex ECM-dependent cell models.

The results obtained herein identify *B2m*, *Tbp*, and *Rplp0* as the most stable reference gene candidates. However, functional validation supported *B2m* and *Rplp0* as the most suitable two-gene normalization panel for the matrix-dependent 3T3-L1 adipogenic model investigated in this study, whereas *Tbp* may be considered an additional complementary reference gene.

## Figures and Tables

**Table 1:** Correlation coefficients (r) and *p*-values based on the BestKeeper calculations.

BestKeeper Index vs.	*18S*	*Actb*	*B2m*	*Gapdh*	*Hmbs*	*Hprt*	*Nono*	*Ppia*	*Rplp0*	*Ywhaz*	*Tbp*	*Pparg*	*Fasn*
*n*	48	48	48	48	48	48	48	48	48	48	48	48	48
coeff. of correlation (r)	0.378	−0.256	0.751	0.312	0.545	0.207	0.371	0.881	0.578	0.874	0.733	0.314	0.355
*p*-value	<0.01	ns	<0.001	<0.05	<0.001	ns	<0.01	<0.001	<0.001	<0.001	<0.001	<0.05	<0.01

**Table 2:** Spearman correlation coefficients (r), raw *p*-values, and Benjamini–Hochberg FDR-adjusted *p*-values for associations between candidate reference genes and adipogenic marker genes.

***Pparg* vs.**	***Gapdh***	***Hprt***	***Hmbs***	***Tbp***	***Ppia***	***Ywhaz***	***Actb***	***B2m***	***18S***	***Rplp0***	***Nono***
*n*	48	48	48	48	48	48	48	48	48	48	48
Spearman (r) correlation	0.417	0.392	0.319	0.314	0.236	0.206	0.188	0.174	0.141	0.093	−0.035
*p*-value	0.003	0.006	0.027	0.030	0.107	0.161	0.200	0.238	0.339	0.529	0.811
FDR-adjusted *p*-value	0.033	0.033	0.099	0.083	0.235	0.295	0.314	0.327	0.414	0.582	0.811
***Fasn*** **vs.**	***Gapdh***	***Hprt***	***Hmbs***	***Tbp***	***18S***	***Ywhaz***	***Ppia***	***Nono***	***B2m***	***Rplp0***	***Actb***
Spearman (r) correlation	0.494	0.394	0.365	0.311	0.271	0.262	0.204	0.190	0.188	0.146	−0.001
*p*-value	0.001	0.006	0.011	0.031	0.063	0.072	0.165	0.197	0.201	0.322	0.998
FDR-adjusted *p*-value	0.011	0.033	0.040	0.085	0.139	0.132	0.259	0.271	0.246	0.354	0.998

**Table 3:** Linear mixed model (LMM) analysis of the effects of Gene, Passage, Matrix, and Day (differentiation stage) on Ct values.

Source	Numerator df	Denominator df	F	Sig.
Intercept	1	39	496,293.45	0.000
Passage	1	39	0.17	0.687
Gene	10	400	4812.23	0.000
Matrix	3	39	9.48	0.000
Day	1	39	28.72	0.000
Gene × Matrix	30	400	3.96	0.000
Gene × Day	10	400	83.13	0.000
Matrix × Day	3	39	1.43	0.248
Gene × Matrix × Day	30	400	6.39	0.000

**Table 4:** GLM-based assessment of matrix- and differentiation stage-dependent variability of candidate reference genes.

Gene	Passage F	*p*	η^2^	Matrix F	*p*	η^2^	Day F	*p*	η^2^	Matrix × Day F	*p*	η^2^
*18S*	1.273	0.266	0.032	6.102	0.002	0.319	0.038	0.847	0.001	10.036	<0.001	0.436
*Actb*	1.605	0.213	0.040	13.532	<0.001	0.510	200.297	<0.001	0.837	4.248	0.011	0.246
*B2m*	0.036	0.851	0.001	2.053	0.122	0.136	35.538	<0.001	0.477	4.126	0.012	0.241
*Gapdh*	3.445	0.071	0.081	1.934	0.140	0.129	8.133	0.007	0.173	3.334	0.029	0.204
*Hmbs*	0.317	0.576	0.008	11.745	<0.001	0.475	93.990	<0.001	0.707	1.409	0.255	0.098
*Hprt*	0.523	0.474	0.013	2.207	0.103	0.145	20.835	<0.001	0.348	5.004	0.005	0.278
*Nono*	6.420	0.015	0.141	1.259	0.302	0.088	75.803	<0.001	0.660	3.296	0.030	0.202
*Ppia*	0.052	0.820	0.001	7.601	<0.001	0.369	195.161	<0.001	0.833	2.026	0.126	0.135
*Rplp0*	0.096	0.758	0.002	3.164	0.035	0.196	20.737	<0.001	0.347	3.615	0.021	0.218
*Tbp*	0.693	0.410	0.017	4.497	0.008	0.257	4.335	0.044	0.100	0.166	0.919	0.013
*Ywhaz*	0.979	0.329	0.024	7.870	<0.001	0.377	127.485	<0.001	0.766	1.640	0.196	0.112

**Table 5:** Descriptive statistics of the candidate reference genes and adipogenic marker genes included in the repeated BestKeeper analysis.

	*Tbp*	*B2m*	*Rplp0*	*Ppia*	*Pparg*	*Fasn*
***n***	48	48	48	48	**48**	**48**
**geo Mean [CP]**	24.92	18.56	18.43	18.23	22.66	24.46
**ar Mean [CP]**	24.92	18.56	18.43	18.24	22.67	24.47
**min [CP]**	23.67	17.89	17.99	16.81	21.48	22.61
**max [CP]**	25.75	19.73	20.04	20.09	24.43	25.93
**std dev [±CP]**	0.23	0.26	0.26	0.67	0.40	0.57
**CV [% CP]**	0.92	1.40	1.41	3.65	1.77	2.32

**Abbreviations:** BK, BestKeeper; CP, crossing point; geo Mean, geometric mean; ar Mean, arithmetic mean; SD, standard deviation; CV, coefficient of variation.

**Table 6:** Linear regression analysis of candidate reference genes and adipogenic marker genes relative to the recalculated BestKeeper index.

	*Tbp* vs. BK	*B2m* vs. BK	*Rplp0* vs. BK	*Ppia* vs. BK	*Pparg* vs. BK	*Fasn* vs. BK
coeff. of corr. [r]	0.635	0.858	0.881	0.936	0.240	0.204
coeff. of det. [R^2^]	0.403	0.736	0.776	0.876	0.058	0.042
intercept [CP]	14.898	3.746	2.375	−16.911	16.512	17.300
slope [CP]	0.505	0.746	0.809	1.770	0.310	0.361
SE [CP]	±0.257	±0.186	±0.181	±0.278	±0.524	±0.724
*p*-value	0.001	0.001	0.001	0.001	0.100	0.165
Power [x-fold]	1.40	1.68	1.74	3.47	1.23	1.28

Abbreviations: BK, BestKeeper; r, Pearson correlation coefficient; R^2^, coefficient of determination; SE, standard error of estimate; CP, crossing point.

**Table 7:** Sequences of selected housekeeping and target genes used in RT-qPCR.

Abbreviation	Full Name	Forward Primer	Reverse Primer	Product Size (bp)
*B2m* NM_009735.3	Beta-2 microglobulin	TGTATGCTATCCAGAAAACCCCT	TTTCAATGTGAGGCGGGTGG	117
*Ppia* NM_008907.2	Peptidylprolyl isomerase A	CTGGACCAAACACAAACGGT	TGCCCGCAAGTCAAAAGAAA	208
*Gapdh* NM_001289726.2	Glyceraldehyde-3-phosphate dehydrogenase	CCCACTCTTCCACCTTCGAT	CTTGCTCAGTGTCCTTGCTG	181
*Ywhaz* NM_001356569.1	Tyrosine 3-monooxygenase/tryptophan 5-monooxygenase activation protein, zeta polypeptide	AGACGGAAGGTGCTGAGAAA	TTGTCATCACCAGCAGCAAC	211
*Hmbs* NM_001110251.1	Hydroxymethylbilane synthase	CCTGAAGGATGTGCCTACCA	CCACTCGAATCACCCTCATCT	175
*Rplp0* NM_007475.5	Ribosomal protein, large, P0	GAAACTGCTGCCTCACATCC	AGGTCTTCTCGGGTCCTAGA	179
*Hprt* NM_013556.2	Hypoxanthine guanine phosphoribosyl transferase	ACAGGCCAGACTTTGTTGGA	ACTTGCGCTCATCTTAGGCT	150
*Actb* NM_007393.5	β-actin	CCTCTATGCCAACACAGTGC	GTACTCCTGCTTGCTGATCC	211
*Tbp* NM_013684.3	TATA-box binding protein	AATTGTACCGCAGCTTCAAAAT	CAGTTGTCCGTGGCTCTCTT	152
*Nono* *NM_001426221.1*	Mus musculus non-POU-domain-containing, octamer-binding protein	AAGGAGAGAGAACAGCCACC	TAACCTGGTGCTCATGACGT	184
*18S* NR_003278.3	18S ribosomal RNA	CGTTCTTAGTTGGTGGAGCG	AACGCCACTTGTCCCTCTAA	127
*Pparg* NM_001127330.2	Peroxisome proliferator-activated receptor gamma	AGGGCGATCTTGACAGGAAA	CGAAACTGGCACCCTTGAAA	164
*Fasn* NM_007988.3	Fatty acid synthase	CTGAAGCCGAACACCTCTGT	GGGAATGTTACACCTTGCTCCT	218

## Data Availability

The original contributions presented in this study are included in the article. Further inquiries can be directed to the corresponding author.

## Supplementary Materials

The following supporting information can be downloaded at: https://www.mdpi.com/article/10.3390/ijms27125268/s1.

## Abbreviations

The following abbreviations are used in this manuscript:

18S	18S ribosomal RNA
Rplp0	Ribosomal protein, large, P0
3T3-L1	Mouse embryonic fibroblast cell line used as an adipogenesis model
Actb	β-actin
B2m	Beta-2 microglobulin
BK	BestKeeper index
cDNA	Complementary deoxyribonucleic acid
CM	Control medium
Ct	Cycle threshold
ΔCt	delta Ct
CV	Coefficient of variation
DMEM	Dulbecco’s Modified Eagle’s Medium
DMSO	Dimethyl sulfoxide
Gapdh	Glyceraldehyde-3-phosphate dehydrogenase
GLM	Generalized linear model
HKGs	Housekeeping genes
Hmbs	Hydroxymethylbilane synthase
Hprt	Hypoxanthine guanine phosphoribosyl transferase
IBMX	3-Isobutyl-1-methylxanthine
IC	Differentiated adipocytes (positive control)
LMM	Linear mixed model
MM	Adipocyte maintenance medium
NC	Undifferentiated control preadipocytes (negative control)
Nono	Non-POU domain-containing octamer-binding protein
PA	Palmitic acid
PBS	Phosphate-buffered saline
Ppia	Peptidylprolyl isomerase A
RNA	Ribonucleic acid
RT-qPCR	Reverse transcription quantitative polymerase chain reaction
SD	Standard deviation
SEM	Standard error of the mean
Tbp	TATA-box binding protein
Ywhaz	Tyrosine 3-monooxygenase/tryptophan 5-monooxygenase activation protein, zeta polypeptide
ORO	Oil Red O stain
