# Supplementary material for: Matrix- and Differentiation Stage-Dependent Variability of Reference Genes: Rethinking Validation Strategies in 3T3-L1 Adipogenic Models
Source: Int J Mol Sci. 2026 Jun 10;27(12):5268. doi: 10.3390/ijms27125268 (PMC13299655; doi:10.3390/ijms27125268)
Supplement: Supplementary file 1 [file ijms-27-05268-s001.zip › ijms-4336871-supplementary.pdf]

# Supplementary Materials

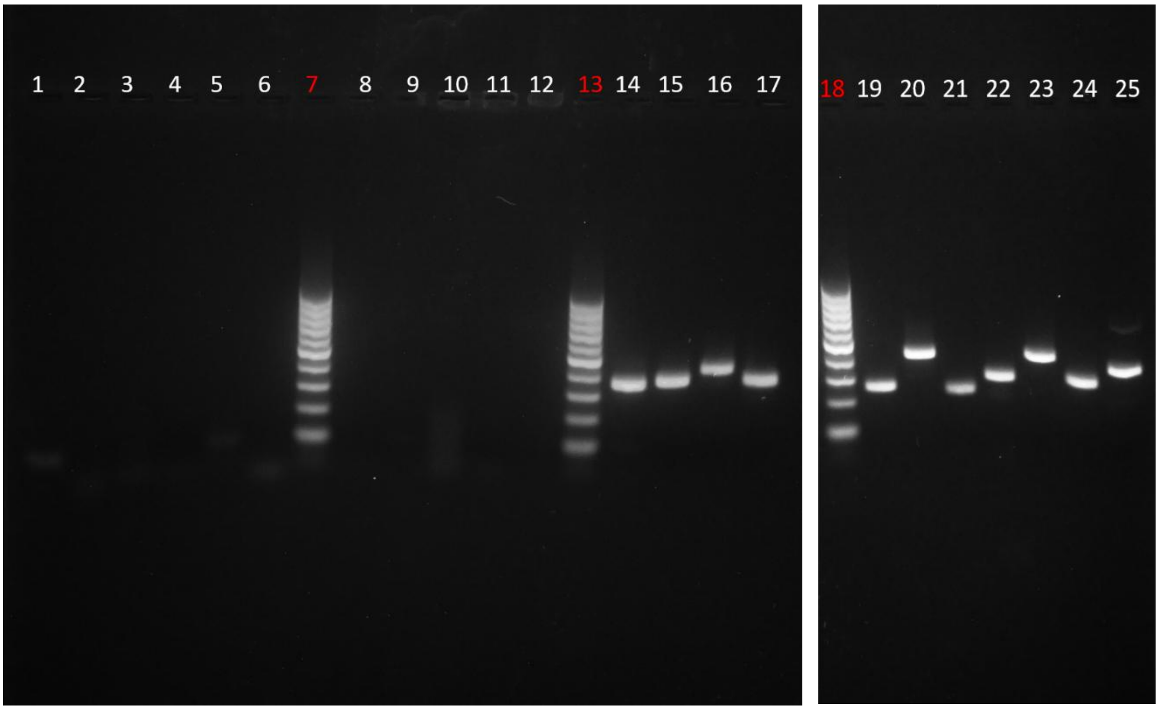


**Figure S1.** Verification of RT− controls and RT+ amplification products for candidate reference genes by agarose gel electrophoresis. PCR products obtained from pooled samples representing all experimental groups were separated on a 1.8% agarose gel prepared in 1× TAE buffer and visualized after electrophoresis (180 V, 40 min). Lanes 1–6 and 8–12 show RT− controls for the candidate reference genes: 18S (lane 1), Actb (lane 2), B2m (lane 3), Hprt (lane 4), Ppia (lane 5), Tbp (lane 6), Nono (lane 8), Hmbs (lane 9), Gapdh (lane 10), Ywhaz (lane 11), and Rplp0 (lane 12). Lanes 14–17 and 19–25 show the corresponding RT+ reactions in the same order. Lanes 7, 13, and 18 contain a 50-bp DNA ladder (50–500 bp), with the 250-bp fragment displayed at higher intensity. The absence of visible amplification products in RT− controls confirmed the lack of detectable genomic DNA contamination.

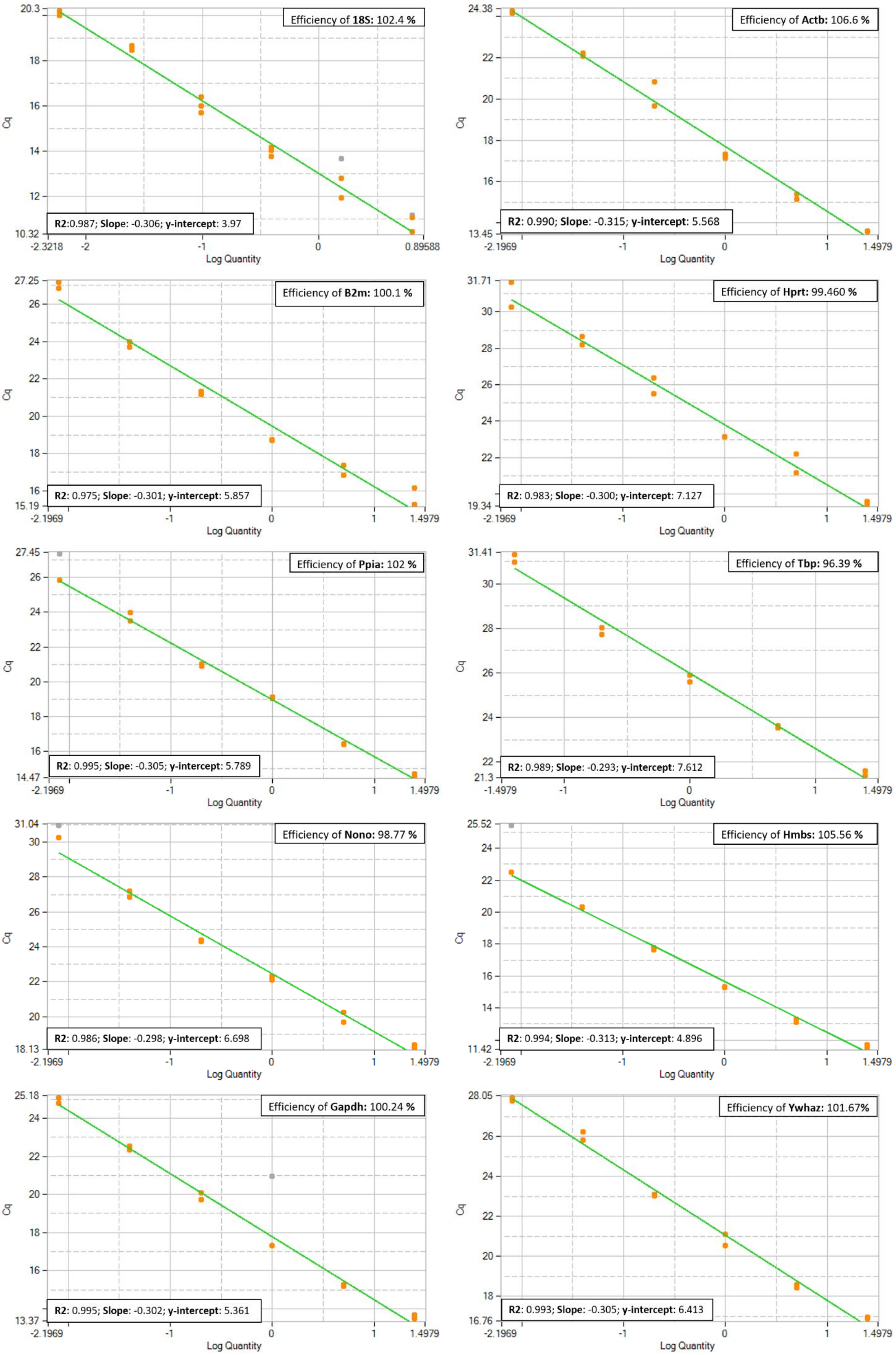
Efficiency of 18S: 102.4 %
R2:0.987; Slope: -0.306; y-intercept: 3.97
Efficiency of Actb: 106.6 %
R2: 0.990; Slope: -0.315; y-intercept: 5.568
Efficiency of B2m: 100.1 %
R2: 0.975; Slope: -0.301; y-intercept: 5.857
Efficiency of Hprt: 99.460 %
R2: 0.983; Slope: -0.300; y-intercept: 7.127
Efficiency of Ppia: 102 %
R2: 0.995; Slope: -0.305; y-intercept: 5.789
Efficiency of Tbp: 96.39 %
R2: 0.989; Slope: -0.293; y-intercept: 7.612
Efficiency of Nono: 98.77 %
R2: 0.986; Slope: -0.298; y-intercept: 6.698
Efficiency of Hmbs: 105.56 %
R2: 0.994; Slope: -0.313; y-intercept: 4.896
Efficiency of Gapdh: 100.24 %
R2: 0.995; Slope: -0.302; y-intercept: 5.361
Efficiency of Ywhaz: 101.67%
R2: 0.993; Slope: -0.305; y-intercept: 6.413
Cq
Log Quantity

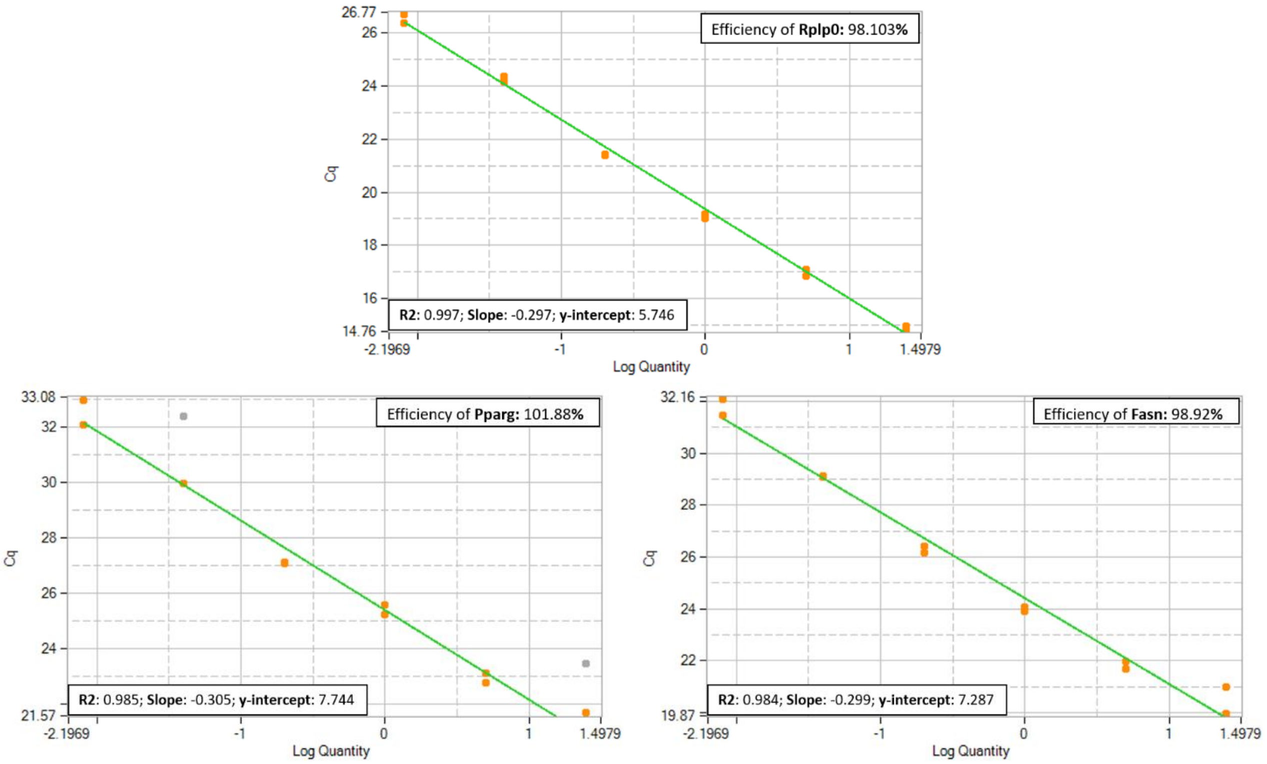


**Figure S2.** Standard curves and amplification efficiencies of candidate reference and target genes used in RT-qPCR analysis. Standard curves were generated for the candidate reference genes 18S, Actb, B2m, Hprt, Ppia, Tbp, Nono, Hmbs, Gapdh, Ywhaz, and Rplp0, as well as for the target genes Fasn and Pparg. A pooled cDNA sample prepared from all experimental samples was subjected to a five-fold serial dilution series (1×, 5×, 25×, 125×, 625×, and 3125×). RT-qPCR reactions were performed in duplicate for all genes, except 18S, which was analyzed in triplicate. Standard curves were generated automatically using the PikoReal software 2.2 (Thermo Scientific) by plotting Cq values against the logarithm of template quantity. Amplification efficiency (E), coefficient of determination ($R^2$), slope, and y-intercept were calculated automatically by the software and are added in each graph. Individual standard-curve plots exported from PikoReal were assembled into a composite figure for comparison of assay performance across all analyzed genes.

**Table S1.** geNorm pairwise variation (Vn/Vn + 1) analysis used to determine the optimal number of reference genes required for reliable normalization.

| Comparison | V value |
|---|---|
| V2/3 | 0.09 |
| V3/4 | 0.062 |
| V4/5 | 0.06 |
| V5/6 | 0.059 |
| V6/7 | 0.069 |
| V7/8 | 0.059 |
| V8/9 | 0.057 |
| V9/10 | 0.081 |
| V10/11 | 0.107 |

**Abbreviations:** Vn/Vn + 1, pairwise variation between normalization factors containing n and n + 1 reference genes.

**Table S2.** Estimates of Fixed Effects Based on Linear Mixed Model Analysis.

| Parameter | Estimates of Fixed Effects[a] | | | | | | |
|---|---|---|---|---|---|---|---|
| | Estimate | Std. Error | df | t | *p*-values | 95% Confidence Interval | |
| | | | | | | Lower Bound | Upper Bound |
| Intercept | 20.50 | 0.14 | 226.18 | 143.46 | 0.00 | 20.22 | 20.78 |
| [Passage=P4] | 0.02 | 0.06 | 39.00 | 0.41 | 0.69 | -0.09 | 0.14 |
| [Passage=P5] | 0[b] | 0 | . | . | . | . | . |
| [Gene=18S] | -2.64 | 0.17 | 400.00 | -15.62 | 0.00 | -2.97 | -2.31 |
| [Gene=Actb] | -2.08 | 0.17 | 400.00 | -12.34 | 0.00 | -2.42 | -1.75 |
| [Gene=B2M] | -1.72 | 0.17 | 400.00 | -10.21 | 0.00 | -2.06 | -1.39 |
| [Gene=Gapdh] | -2.85 | 0.17 | 400.00 | -16.85 | 0.00 | -3.18 | -2.51 |
| [Gene=Hmbs] | 4.48 | 0.17 | 400.00 | 26.51 | 0.00 | 4.15 | 4.81 |
| [Gene=Hprt] | 2.63 | 0.17 | 400.00 | 15.56 | 0.00 | 2.30 | 2.96 |
| [Gene=Nono] | 1.99 | 0.17 | 400.00 | 11.80 | 0.00 | 1.66 | 2.33 |
| [Gene=Ppia] | -1.75 | 0.17 | 400.00 | -10.34 | 0.00 | -2.08 | -1.41 |
| [Gene=Rplp0] | -2.10 | 0.17 | 400.00 | -12.43 | 0.00 | -2.43 | -1.77 |
| [Gene=Tbp] | 4.52 | 0.17 | 400.00 | 26.75 | 0.00 | 4.19 | 4.85 |
| [Gene=Ywhaz] | 0[b] | 0 | . | . | . | . | . |
| [Matrix=Collagen] | -0.31 | 0.20 | 248.46 | -1.55 | 0.12 | -0.70 | 0.08 |
| [Matrix=Gelatin peptone] | -0.02 | 0.20 | 248.46 | -0.08 | 0.94 | -0.41 | 0.37 |
| [Matrix=Matrigel] | 0.33 | 0.20 | 248.46 | 1.66 | 0.10 | -0.06 | 0.72 |
| [Matrix=TCP] | 0[b] | 0 | . | . | . | . | . |
| [Day=7] | -0.69 | 0.20 | 248.46 | -3.48 | 0.00 | -1.08 | -0.30 |
| [Day=14] | 0[b] | 0 | . | . | . | . | . |
| [Gene=18S] * [Matrix=Collagen] | -1.21 | 0.24 | 400.00 | -5.04 | 0.00 | -1.67 | -0.74 |
| [Gene=18S] * [Matrix=Gelatin peptone] | -1.59 | 0.24 | 400.00 | -6.67 | 0.00 | -2.06 | -1.12 |
| [Gene=18S] * [Matrix=Matrigel] | -1.90 | 0.24 | 400.00 | -7.95 | 0.00 | -2.37 | -1.43 |
| [Gene=18S] * [Matrix=TCP] | 0[b] | 0 | . | . | . | . | . |
| [Gene=Actb] * [Matrix=Collagen] | -0.90 | 0.24 | 400.00 | -3.77 | 0.00 | -1.37 | -0.43 |
| [Gene=Actb] * [Matrix=Gelatin peptone] | -1.07 | 0.24 | 400.00 | -4.47 | 0.00 | -1.54 | -0.60 |
| [Gene=Actb] * [Matrix=Matrigel] | -0.54 | 0.24 | 400.00 | -2.27 | 0.02 | -1.01 | -0.07 |
| [Gene=Actb] * [Matrix=TCP] | 0[b] | 0 | . | . | . | . | . |
| [Gene=B2M] * [Matrix=Collagen] | 0.06 | 0.24 | 400.00 | 0.23 | 0.82 | -0.41 | 0.52 |
| [Gene=B2M] * [Matrix=Gelatin peptone] | -0.06 | 0.24 | 400.00 | -0.23 | 0.82 | -0.53 | 0.41 |
| [Gene=B2M] * [Matrix=Matrigel] | -0.05 | 0.24 | 400.00 | -0.22 | 0.83 | -0.52 | 0.42 |
| [Gene=B2M] * [Matrix=TCP] | 0[b] | 0 | . | . | . | . | . |
| [Gene=Gapdh] * [Matrix=Collagen] | -0.20 | 0.24 | 400.00 | -0.82 | 0.41 | -0.67 | 0.27 |
| [Gene=Gapdh] * [Matrix=Gelatin peptone] | -0.18 | 0.24 | 400.00 | -0.73 | 0.46 | -0.64 | 0.29 |
| [Gene=Gapdh] * [Matrix=Matrigel] | -0.50 | 0.24 | 400.00 | -2.11 | 0.04 | -0.97 | -0.03 |
| [Gene=Gapdh] * [Matrix=TCP] | 0[b] | 0 | . | . | . | . | . |
| [Gene=Hmbs] * [Matrix=Collagen] | 0.18 | 0.24 | 400.00 | 0.74 | 0.46 | -0.29 | 0.65 |
| [Gene=Hmbs] * [Matrix=Gelatin peptone] | 0.22 | 0.24 | 400.00 | 0.92 | 0.36 | -0.25 | 0.69 |
| [Gene=Hmbs] * [Matrix=Matrigel] | 0.17 | 0.24 | 400.00 | 0.71 | 0.48 | -0.30 | 0.64 |
| [Gene=Hmbs] * [Matrix=TCP] | 0[b] | 0 | . | . | . | . | . |
| [Gene=Hprt] * [Matrix=Collagen] | 0.14 | 0.24 | 400.00 | 0.59 | 0.56 | -0.33 | 0.61 |
| [Gene=Hprt] * [Matrix=Gelatin peptone] | 0.24 | 0.24 | 400.00 | 1.01 | 0.31 | -0.23 | 0.71 |
| [Gene=Hprt] * [Matrix=Matrigel] | -0.18 | 0.24 | 400.00 | -0.73 | 0.46 | -0.64 | 0.29 |
| [Gene=Hprt] * [Matrix=TCP] | 0[b] | 0 | . | . | . | . | . |
| [Gene=Nono] * [Matrix=Collagen] | 0.45 | 0.24 | 400.00 | 1.89 | 0.06 | -0.02 | 0.92 |
| [Gene=Nono] * [Matrix=Gelatin peptone] | 0.13 | 0.24 | 400.00 | 0.56 | 0.58 | -0.34 | 0.60 |
| [Gene=Nono] * [Matrix=Matrigel] | -0.44 | 0.24 | 400.00 | -1.82 | 0.07 | -0.91 | 0.03 |
| [Gene=Nono] * [Matrix=TCP] | 0[b] | 0 | . | . | . | . | . |
| [Gene=Ppia] * [Matrix=Collagen] | 0.13 | 0.24 | 400.00 | 0.52 | 0.60 | -0.34 | 0.59 |
| [Gene=Ppia] * [Matrix=Gelatin peptone] | 0.16 | 0.24 | 400.00 | 0.65 | 0.51 | -0.31 | 0.63 |
| [Gene=Ppia] * [Matrix=Matrigel] | 0.27 | 0.24 | 400.00 | 1.14 | 0.26 | -0.20 | 0.74 |
| [Gene=Ppia] * [Matrix=TCP] | 0[b] | 0 | . | . | . | . | . |
| [Gene=Rplp0] * [Matrix=Collagen] | 0.40 | 0.24 | 400.00 | 1.67 | 0.10 | -0.07 | 0.87 |
| [Gene=Rplp0] * [Matrix=Gelatin peptone] | 0.10 | 0.24 | 400.00 | 0.41 | 0.68 | -0.37 | 0.57 |
| [Gene=Rplp0] * [Matrix=Matrigel] | 0.33 | 0.24 | 400.00 | 1.38 | 0.17 | -0.14 | 0.80 |
| [Gene=Rplp0] * [Matrix=TCP] | 0[b] | 0 | . | . | . | . | . |

| Parameter | Estimates of Fixed Effects[a] | | | | | | |
|---|---|---|---|---|---|---|---|
| | Estimate | Std. Error | df | t | *p*-values | 95% Confidence Interval | |
| | | | | | | Lower Bound | Upper Bound |
| [Gene=Tbp] * [Matrix=Collagen] | 0.15 | 0.24 | 400.00 | 0.61 | 0.54 | -0.32 | 0.62 |
| [Gene=Tbp] * [Matrix=Gelatin peptone] | -0.17 | 0.24 | 400.00 | -0.72 | 0.47 | -0.64 | 0.30 |
| [Gene=Tbp] * [Matrix=Matrigel] | -0.07 | 0.24 | 400.00 | -0.29 | 0.77 | -0.54 | 0.40 |
| [Gene=Tbp] * [Matrix=TCP] | 0[b] | 0 | . | . | . | . | . |
| [Gene=Ywhaz] * [Matrix=Collagen] | 0[b] | 0 | . | . | . | . | . |
| [Gene=Ywhaz] * [Matrix=Gelatin peptone] | 0[b] | 0 | . | . | . | . | . |
| [Gene=Ywhaz] * [Matrix=Matrigel] | 0[b] | 0 | . | . | . | . | . |
| [Gene=Ywhaz] * [Matrix=TCP] | 0[b] | 0 | . | . | . | . | . |
| [Gene=18S] * [Day=7] | -0.65 | 0.24 | 400.00 | -2.74 | 0.01 | -1.12 | -0.18 |
| [Gene=18S] * [Day=14] | 0[b] | 0 | . | . | . | . | . |
| [Gene=Actb] * [Day=7] | 2.03 | 0.24 | 400.00 | 8.48 | 0.00 | 1.56 | 2.50 |
| [Gene=Actb] * [Day=14] | 0[b] | 0 | . | . | . | . | . |
| [Gene=B2M] * [Day=7] | 0.33 | 0.24 | 400.00 | 1.36 | 0.17 | -0.14 | 0.80 |
| [Gene=B2M] * [Day=14] | 0[b] | 0 | . | . | . | . | . |
| [Gene=Gapdh] * [Day=7] | 0.48 | 0.24 | 400.00 | 2.03 | 0.04 | 0.01 | 0.95 |
| [Gene=Gapdh] * [Day=14] | 0[b] | 0 | . | . | . | . | . |
| [Gene=Hmbs] * [Day=7] | 0.17 | 0.24 | 400.00 | 0.73 | 0.47 | -0.30 | 0.64 |
| [Gene=Hmbs] * [Day=14] | 0[b] | 0 | . | . | . | . | . |
| [Gene=Hprt] * [Day=7] | 0.41 | 0.24 | 400.00 | 1.73 | 0.08 | -0.06 | 0.88 |
| [Gene=Hprt] * [Day=14] | 0[b] | 0 | . | . | . | . | . |
| [Gene=Nono] * [Day=7] | -0.35 | 0.24 | 400.00 | -1.47 | 0.14 | -0.82 | 0.12 |
| [Gene=Nono] * [Day=14] | 0[b] | 0 | . | . | . | . | . |
| [Gene=Ppia] * [Day=7] | -0.34 | 0.24 | 400.00 | -1.40 | 0.16 | -0.80 | 0.13 |
| [Gene=Ppia] * [Day=14] | 0[b] | 0 | . | . | . | . | . |
| [Gene=Rplp0] * [Day=7] | 0.56 | 0.24 | 400.00 | 2.32 | 0.02 | 0.09 | 1.02 |
| [Gene=Rplp0] * [Day=14] | 0[b] | 0 | . | . | . | . | . |
| [Gene=Tbp] * [Day=7] | 0.48 | 0.24 | 400.00 | 2.00 | 0.05 | 0.01 | 0.95 |
| [Gene=Tbp] * [Day=14] | 0[b] | 0 | . | . | . | . | . |
| [Gene=Ywhaz] * [Day=7] | 0[b] | 0 | . | . | . | . | . |
| [Gene=Ywhaz] * [Day=14] | 0[b] | 0 | . | . | . | . | . |
| [Matrix=Collagen] * [Day=7] | -0.04 | 0.28 | 248.46 | -0.15 | 0.88 | -0.59 | 0.51 |
| [Matrix=Collagen] * [Day=14] | 0[b] | 0 | . | . | . | . | . |
| [Matrix=Gelatin peptone] * [Day=7] | -0.39 | 0.28 | 248.46 | -1.40 | 0.16 | -0.94 | 0.16 |
| [Matrix=Gelatin peptone] * [Day=14] | 0[b] | 0 | . | . | . | . | . |
| [Matrix=Matrigel] * [Day=7] | -0.33 | 0.28 | 248.46 | -1.19 | 0.24 | -0.88 | 0.22 |
| [Matrix=Matrigel] * [Day=14] | 0[b] | 0 | . | . | . | . | . |
| [Matrix=TCP] * [Day=7] | 0[b] | 0 | . | . | . | . | . |
| [Matrix=TCP] * [Day=14] | 0[b] | 0 | . | . | . | . | . |
| [Gene=18S] * [Matrix=Collagen] * [Day=7] | 1.48 | 0.34 | 400.00 | 4.39 | 0.00 | 0.82 | 2.15 |
| [Gene=18S] * [Matrix=Collagen] * [Day=14] | 0[b] | 0 | . | . | . | . | . |
| [Gene=18S] * [Matrix=Gelatin peptone] * [Day=7] | 1.91 | 0.34 | 400.00 | 5.65 | 0.00 | 1.24 | 2.57 |
| [Gene=18S] * [Matrix=Gelatin peptone] * [Day=14] | 0[b] | 0 | . | . | . | . | . |
| [Gene=18S] * [Matrix=Matrigel] * [Day=7] | 2.87 | 0.34 | 400.00 | 8.49 | 0.00 | 2.21 | 3.53 |
| [Gene=18S] * [Matrix=Matrigel] * [Day=14] | 0[b] | 0 | . | . | . | . | . |
| [Gene=18S] * [Matrix=TCP] * [Day=7] | 0[b] | 0 | . | . | . | . | . |
| [Gene=18S] * [Matrix=TCP] * [Day=14] | 0[b] | 0 | . | . | . | . | . |
| [Gene=Actb] * [Matrix=Collagen] * [Day=7] | 0.83 | 0.34 | 400.00 | 2.45 | 0.02 | 0.16 | 1.49 |
| [Gene=Actb] * [Matrix=Collagen] * [Day=14] | 0[b] | 0 | . | . | . | . | . |
| [Gene=Actb] * [Matrix=Gelatin peptone] * [Day=7] | 0.83 | 0.34 | 400.00 | 2.45 | 0.02 | 0.16 | 1.49 |
| [Gene=Actb] * [Matrix=Gelatin peptone] * [Day=14] | 0[b] | 0 | . | . | . | . | . |
| [Gene=Actb] * [Matrix=Matrigel] * [Day=7] | 0.08 | 0.34 | 400.00 | 0.24 | 0.81 | -0.58 | 0.74 |
| [Gene=Actb] * [Matrix=Matrigel] * [Day=14] | 0[b] | 0 | . | . | . | . | . |
| [Gene=Actb] * [Matrix=TCP] * [Day=7] | 0[b] | 0 | . | . | . | . | . |
| [Gene=Actb] * [Matrix=TCP] * [Day=14] | 0[b] | 0 | . | . | . | . | . |
| [Gene=B2M] * [Matrix=Collagen] * [Day=7] | 0.34 | 0.34 | 400.00 | 0.99 | 0.32 | -0.33 | 1.00 |
| [Gene=B2M] * [Matrix=Collagen] * [Day=14] | 0[b] | 0 | . | . | . | . | . |
| [Gene=B2M] * [Matrix=Gelatin peptone] * [Day=7] | 0.21 | 0.34 | 400.00 | 0.61 | 0.54 | -0.46 | 0.87 |
| [Gene=B2M] * [Matrix=Gelatin peptone] * [Day=14] | 0[b] | 0 | . | . | . | . | . |

| Parameter | Estimates of Fixed Effects[a] | | | | | | |
|---|---|---|---|---|---|---|---|
| | Estimate | Std. Error | df | t | *p*-values | 95% Confidence Interval | |
| | | | | | | Lower Bound | Upper Bound |
| [Gene=B2M] * [Matrix=Matrigel] * [Day=7] | -0.08 | 0.34 | 400.00 | -0.24 | 0.81 | -0.75 | 0.58 |
| [Gene=B2M] * [Matrix=Matrigel] * [Day=14] | 0[b] | 0 | . | . | . | . | . |
| [Gene=B2M] * [Matrix=TCP] * [Day=7] | 0[b] | 0 | . | . | . | . | . |
| [Gene=B2M] * [Matrix=TCP] * [Day=14] | 0[b] | 0 | . | . | . | . | . |
| [Gene=Gapdh] * [Matrix=Collagen] * [Day=7] | 0.73 | 0.34 | 400.00 | 2.16 | 0.03 | 0.07 | 1.40 |
| [Gene=Gapdh] * [Matrix=Collagen] * [Day=14] | 0[b] | 0 | . | . | . | . | . |
| [Gene=Gapdh] * [Matrix=Gelatin peptone] * [Day=7] | 0.93 | 0.34 | 400.00 | 2.76 | 0.01 | 0.27 | 1.60 |
| [Gene=Gapdh] * [Matrix=Gelatin peptone] * [Day=14] | 0[b] | 0 | . | . | . | . | . |
| [Gene=Gapdh] * [Matrix=Matrigel] * [Day=7] | 0.86 | 0.34 | 400.00 | 2.55 | 0.01 | 0.20 | 1.53 |
| [Gene=Gapdh] * [Matrix=Matrigel] * [Day=14] | 0[b] | 0 | . | . | . | . | . |
| [Gene=Gapdh] * [Matrix=TCP] * [Day=7] | 0[b] | 0 | . | . | . | . | . |
| [Gene=Gapdh] * [Matrix=TCP] * [Day=14] | 0[b] | 0 | . | . | . | . | . |
| [Gene=Hmbs] * [Matrix=Collagen] * [Day=7] | -0.16 | 0.34 | 400.00 | -0.47 | 0.64 | -0.82 | 0.51 |
| [Gene=Hmbs] * [Matrix=Collagen] * [Day=14] | 0[b] | 0 | . | . | . | . | . |
| [Gene=Hmbs] * [Matrix=Gelatin peptone] * [Day=7] | -0.04 | 0.34 | 400.00 | -0.13 | 0.90 | -0.71 | 0.62 |
| [Gene=Hmbs] * [Matrix=Gelatin peptone] * [Day=14] | 0[b] | 0 | . | . | . | . | . |
| [Gene=Hmbs] * [Matrix=Matrigel] * [Day=7] | 0.11 | 0.34 | 400.00 | 0.33 | 0.74 | -0.55 | 0.78 |
| [Gene=Hmbs] * [Matrix=Matrigel] * [Day=14] | 0[b] | 0 | . | . | . | . | . |
| [Gene=Hmbs] * [Matrix=TCP] * [Day=7] | 0[b] | 0 | . | . | . | . | . |
| [Gene=Hmbs] * [Matrix=TCP] * [Day=14] | 0[b] | 0 | . | . | . | . | . |
| [Gene=Hprt] * [Matrix=Collagen] * [Day=7] | 0.21 | 0.34 | 400.00 | 0.61 | 0.54 | -0.46 | 0.87 |
| [Gene=Hprt] * [Matrix=Collagen] * [Day=14] | 0[b] | 0 | . | . | . | . | . |
| [Gene=Hprt] * [Matrix=Gelatin peptone] * [Day=7] | -0.32 | 0.34 | 400.00 | -0.94 | 0.35 | -0.98 | 0.35 |
| [Gene=Hprt] * [Matrix=Gelatin peptone] * [Day=14] | 0[b] | 0 | . | . | . | . | . |
| [Gene=Hprt] * [Matrix=Matrigel] * [Day=7] | 0.36 | 0.34 | 400.00 | 1.07 | 0.29 | -0.30 | 1.03 |
| [Gene=Hprt] * [Matrix=Matrigel] * [Day=14] | 0[b] | 0 | . | . | . | . | . |
| [Gene=Hprt] * [Matrix=TCP] * [Day=7] | 0[b] | 0 | . | . | . | . | . |
| [Gene=Hprt] * [Matrix=TCP] * [Day=14] | 0[b] | 0 | . | . | . | . | . |
| [Gene=Nono] * [Matrix=Collagen] * [Day=7] | 0.12 | 0.34 | 400.00 | 0.35 | 0.73 | -0.55 | 0.78 |
| [Gene=Nono] * [Matrix=Collagen] * [Day=14] | 0[b] | 0 | . | . | . | . | . |
| [Gene=Nono] * [Matrix=Gelatin peptone] * [Day=7] | 0.19 | 0.34 | 400.00 | 0.56 | 0.57 | -0.47 | 0.85 |
| [Gene=Nono] * [Matrix=Gelatin peptone] * [Day=14] | 0[b] | 0 | . | . | . | . | . |
| [Gene=Nono] * [Matrix=Matrigel] * [Day=7] | 1.01 | 0.34 | 400.00 | 2.97 | 0.00 | 0.34 | 1.67 |
| [Gene=Nono] * [Matrix=Matrigel] * [Day=14] | 0[b] | 0 | . | . | . | . | . |
| [Gene=Nono] * [Matrix=TCP] * [Day=7] | 0[b] | 0 | . | . | . | . | . |
| [Gene=Nono] * [Matrix=TCP] * [Day=14] | 0[b] | 0 | . | . | . | . | . |
| [Gene=Ppia] * [Matrix=Collagen] * [Day=7] | -0.12 | 0.34 | 400.00 | -0.35 | 0.73 | -0.78 | 0.55 |
| [Gene=Ppia] * [Matrix=Collagen] * [Day=14] | 0[b] | 0 | . | . | . | . | . |
| [Gene=Ppia] * [Matrix=Gelatin peptone] * [Day=7] | -0.17 | 0.34 | 400.00 | -0.50 | 0.62 | -0.83 | 0.50 |
| [Gene=Ppia] * [Matrix=Gelatin peptone] * [Day=14] | 0[b] | 0 | . | . | . | . | . |
| [Gene=Ppia] * [Matrix=Matrigel] * [Day=7] | -0.18 | 0.34 | 400.00 | -0.53 | 0.60 | -0.84 | 0.49 |
| [Gene=Ppia] * [Matrix=Matrigel] * [Day=14] | 0[b] | 0 | . | . | . | . | . |
| [Gene=Ppia] * [Matrix=TCP] * [Day=7] | 0[b] | 0 | . | . | . | . | . |
| [Gene=Ppia] * [Matrix=TCP] * [Day=14] | 0[b] | 0 | . | . | . | . | . |
| [Gene=Rplp0] * [Matrix=Collagen] * [Day=7] | -0.04 | 0.34 | 400.00 | -0.12 | 0.91 | -0.70 | 0.63 |
| [Gene=Rplp0] * [Matrix=Collagen] * [Day=14] | 0[b] | 0 | . | . | . | . | . |
| [Gene=Rplp0] * [Matrix=Gelatin peptone] * [Day=7] | 0.19 | 0.34 | 400.00 | 0.56 | 0.58 | -0.48 | 0.85 |
| [Gene=Rplp0] * [Matrix=Gelatin peptone] * [Day=14] | 0[b] | 0 | . | . | . | . | . |
| [Gene=Rplp0] * [Matrix=Matrigel] * [Day=7] | -0.38 | 0.34 | 400.00 | -1.12 | 0.26 | -1.04 | 0.29 |
| [Gene=Rplp0] * [Matrix=Matrigel] * [Day=14] | 0[b] | 0 | . | . | . | . | . |
| [Gene=Rplp0] * [Matrix=TCP] * [Day=7] | 0[b] | 0 | . | . | . | . | . |
| [Gene=Rplp0] * [Matrix=TCP] * [Day=14] | 0[b] | 0 | . | . | . | . | . |
| [Gene=Tbp] * [Matrix=Collagen] * [Day=7] | 0.17 | 0.34 | 400.00 | 0.51 | 0.61 | -0.49 | 0.84 |
| [Gene=Tbp] * [Matrix=Collagen] * [Day=14] | 0[b] | 0 | . | . | . | . | . |
| [Gene=Tbp] * [Matrix=Gelatin peptone] * [Day=7] | 0.43 | 0.34 | 400.00 | 1.26 | 0.21 | -0.24 | 1.09 |
| [Gene=Tbp] * [Matrix=Gelatin peptone] * [Day=14] | 0[b] | 0 | . | . | . | . | . |
| [Gene=Tbp] * [Matrix=Matrigel] * [Day=7] | 0.30 | 0.34 | 400.00 | 0.90 | 0.37 | -0.36 | 0.97 |
| [Gene=Tbp] * [Matrix=Matrigel] * [Day=14] | 0[b] | 0 | . | . | . | . | . |

| Parameter | Estimates of Fixed Effects[a] | | | | | | |
|---|---|---|---|---|---|---|---|
| | Estimate | Std. Error | df | t | *p*-values | 95% Confidence Interval | |
| | | | | | | Lower Bound | Upper Bound |
| [Gene=Tbp] * [Matrix=TCP] * [Day=7] | 0[b] | 0 | . | . | . | . | . |
| [Gene=Tbp] * [Matrix=TCP] * [Day=14] | 0[b] | 0 | . | . | . | . | . |
| [Gene=Ywhaz] * [Matrix=Collagen] * [Day=7] | 0[b] | 0 | . | . | . | . | . |
| [Gene=Ywhaz] * [Matrix=Collagen] * [Day=14] | 0[b] | 0 | . | . | . | . | . |
| [Gene=Ywhaz] * [Matrix=Gelatin peptone] * [Day=7] | 0[b] | 0 | . | . | . | . | . |
| [Gene=Ywhaz] * [Matrix=Gelatin peptone] * [Day=14] | 0[b] | 0 | . | . | . | . | . |
| [Gene=Ywhaz] * [Matrix=Matrigel] * [Day=7] | 0[b] | 0 | . | . | . | . | . |
| [Gene=Ywhaz] * [Matrix=Matrigel] * [Day=14] | 0[b] | 0 | . | . | . | . | . |
| [Gene=Ywhaz] * [Matrix=TCP] * [Day=7] | 0[b] | 0 | . | . | . | . | . |
| [Gene=Ywhaz] * [Matrix=TCP] * [Day=14] | 0[b] | 0 | . | . | . | . | . |

**Note:** Reference categories were Passage P5, Ywhaz, TCP, and Day 14. Fixed effect estimates represent the direction and magnitude of the effect relative to the corresponding reference category.

**Table S3.** Raw quantification cycle (Cq) values of candidate reference genes and target genes (Fasn and Pparg) in 3T3-L1 cells.

| SampleID | 18S | Actb | B2m | Gapdh | Hmbs | Hprt | Nono | Ppia | Rplp0 | Ywhaz | Tbp | Pparg | Fasn |
|---|---|---|---|---|---|---|---|---|---|---|---|---|---|
| **TCP_7_R1_P4** | 16.13 | 19.13 | 18.34 | 17.11 | 24.44 | 22.26 | 21.38 | 17.90 | 18.17 | 19.72 | 24.94 | 22.31 | 23.21 |
| **TCP_7_R2_P4** | 16.52 | 19.25 | 18.16 | 17.48 | 24.25 | 22.94 | 21.44 | 17.82 | 18.29 | 19.86 | 24.77 | 22.52 | 24.79 |
| **TCP_7_R3_P4** | 16.29 | 19.11 | 18.71 | 17.61 | 24.72 | 22.88 | 22.02 | 17.95 | 18.33 | 20.04 | 24.96 | 22.27 | 25.26 |
| **TCP_7_R4_P5** | 17.45 | 19.52 | 18.53 | 17.59 | 24.31 | 23.16 | 21.52 | 17.83 | 18.24 | 19.81 | 25.10 | 23.11 | 24.86 |
| **TCP_7_R5_P5** | 16.58 | 20.54 | 18.27 | 17.70 | 24.84 | 23.09 | 21.32 | 18.00 | 18.55 | 19.96 | 24.30 | 22.77 | 24.04 |
| **TCP_7_R6_P5** | 16.25 | 21.08 | 18.56 | 17.31 | 24.32 | 22.90 | 21.14 | 16.98 | 18.12 | 19.58 | 24.87 | 24.43 | 23.86 |
| **TCP_14_R1_P4** | 19.62 | 18.36 | 18.83 | 17.46 | 24.91 | 23.19 | 22.14 | 18.87 | 18.67 | 20.45 | 25.02 | 22.88 | 24.83 |
| **TCP_14_R2_P4** | 18.26 | 18.40 | 18.66 | 17.35 | 24.66 | 23.21 | 23.47 | 18.77 | 18.13 | 20.59 | 25.00 | 23.05 | 24.28 |
| **TCP_14_R3_P4** | 18.38 | 18.82 | 18.81 | 17.87 | 25.12 | 23.10 | 22.35 | 18.57 | 18.14 | 20.36 | 24.89 | 22.41 | 24.94 |
| **TCP_14_R4_P5** | 16.67 | 18.54 | 18.77 | 17.67 | 25.22 | 23.12 | 22.34 | 18.63 | 18.59 | 20.53 | 25.03 | 22.13 | 24.93 |
| **TCP_14_R5_P5** | 16.97 | 18.29 | 18.78 | 17.60 | 25.00 | 23.20 | 22.54 | 18.93 | 18.60 | 20.64 | 25.10 | 22.58 | 24.03 |
| **TCP_14_R6_P5** | 17.36 | 18.19 | 18.91 | 18.08 | 25.06 | 23.06 | 22.23 | 18.85 | 18.38 | 20.54 | 25.17 | 22.73 | 25.25 |
| **Matrigel_7_R1_P4** | 16.22 | 19.21 | 18.46 | 17.59 | 24.40 | 22.65 | 21.76 | 17.62 | 17.99 | 19.48 | 24.83 | 23.06 | 24.38 |
| **Matrigel_7_R2_P4** | 17.28 | 19.30 | 18.37 | 17.57 | 24.95 | 22.90 | 22.03 | 18.02 | 18.23 | 19.91 | 25.12 | 22.71 | 23.35 |
| **Matrigel_7_R3_P4** | 18.23 | 19.33 | 18.35 | 17.71 | 24.42 | 23.19 | 22.52 | 16.95 | 18.33 | 20.02 | 25.06 | 22.49 | 25.53 |
| **Matrigel_7_R4_P5** | 18.25 | 19.15 | 17.92 | 17.71 | 24.95 | 23.17 | 22.34 | 18.22 | 18.18 | 19.94 | 25.10 | 23.12 | 24.63 |
| **Matrigel_7_R5_P5** | 17.87 | 19.37 | 18.17 | 18.07 | 24.98 | 23.02 | 22.04 | 18.09 | 18.29 | 19.80 | 25.17 | 23.18 | 25.63 |
| **Matrigel_7_R6_P5** | 17.17 | 19.47 | 18.50 | 18.27 | 24.84 | 23.39 | 21.52 | 18.12 | 18.36 | 19.79 | 25.04 | 23.38 | 25.53 |
| **Matrigel_14_R1_P4** | 16.35 | 18.20 | 19.55 | 17.94 | 26.04 | 23.63 | 23.03 | 20.02 | 19.60 | 21.56 | 25.75 | 23.33 | 25.28 |
| **Matrigel_14_R2_P4** | 17.42 | 18.21 | 19.73 | 17.96 | 26.20 | 24.08 | 23.01 | 20.09 | 20.04 | 21.67 | 25.62 | 23.77 | 25.30 |
| **Matrigel_14_R3_P4** | 15.82 | 18.41 | 18.80 | 17.19 | 25.11 | 22.85 | 22.21 | 19.04 | 18.72 | 20.55 | 24.99 | 22.60 | 24.42 |
| **Matrigel_14_R4_P5** | 16.22 | 18.18 | 18.80 | 17.49 | 25.18 | 23.00 | 22.06 | 19.02 | 18.80 | 20.48 | 25.08 | 22.96 | 24.04 |
| **Matrigel_14_R5_P5** | 16.07 | 18.17 | 18.89 | 17.29 | 25.22 | 23.43 | 22.02 | 18.89 | 18.49 | 20.49 | 25.17 | 22.97 | 24.41 |
| **Matrigel_14_R6_P5** | 15.96 | 18.15 | 18.65 | 17.10 | 25.21 | 22.81 | 22.07 | 19.17 | 18.79 | 20.32 | 25.16 | 22.59 | 24.79 |
| **Collagen_7_R1_P4** | 16.39 | 19.41 | 18.48 | 17.92 | 24.23 | 22.26 | 21.81 | 17.51 | 18.70 | 19.49 | 24.85 | 22.41 | 24.21 |
| **Collagen_7_R2_P4** | 16.81 | 19.35 | 18.22 | 17.47 | 24.22 | 22.94 | 21.58 | 17.31 | 18.06 | 19.49 | 24.58 | 21.81 | 24.11 |
| **Collagen_7_R3_P4** | 16.84 | 19.31 | 18.23 | 17.60 | 24.03 | 22.88 | 21.85 | 17.49 | 18.37 | 19.55 | 24.87 | 21.73 | 23.26 |
| **Collagen_7_R4_P5** | 16.32 | 19.39 | 18.65 | 17.55 | 23.94 | 23.16 | 22.20 | 17.29 | 18.09 | 19.48 | 24.63 | 22.23 | 24.20 |
| **Collagen_7_R5_P5** | 16.39 | 19.32 | 18.89 | 17.46 | 24.35 | 23.09 | 21.64 | 17.61 | 18.44 | 19.66 | 25.11 | 22.41 | 24.38 |
| **Collagen_7_R6_P5** | 16.03 | 19.31 | 18.36 | 17.91 | 24.14 | 22.90 | 21.07 | 17.21 | 18.11 | 19.22 | 24.72 | 23.39 | 24.47 |
| **Collagen_14_R1_P4** | 16.06 | 17.22 | 18.68 | 17.10 | 24.76 | 23.12 | 23.50 | 18.58 | 18.16 | 20.14 | 24.78 | 21.95 | 22.61 |
| **Collagen_14_R2_P4** | 16.63 | 17.23 | 18.52 | 16.55 | 24.67 | 22.90 | 22.43 | 18.51 | 18.63 | 19.97 | 25.03 | 21.48 | 24.00 |
| **Collagen_14_R3_P4** | 16.62 | 17.58 | 18.58 | 17.57 | 25.23 | 23.56 | 22.91 | 19.00 | 18.88 | 20.75 | 25.12 | 22.44 | 25.15 |
| **Collagen_14_R4_P5** | 16.23 | 17.18 | 18.57 | 17.09 | 24.72 | 22.75 | 22.21 | 18.33 | 18.35 | 20.04 | 24.96 | 22.08 | 23.42 |
| **Collagen_14_R5_P5** | 16.37 | 17.24 | 18.34 | 17.15 | 24.90 | 22.94 | 22.18 | 18.41 | 18.49 | 20.12 | 25.02 | 22.08 | 22.89 |
| **Collagen_14_R6_P5** | 16.28 | 16.90 | 18.56 | 17.54 | 24.91 | 22.61 | 22.69 | 18.69 | 18.55 | 20.24 | 24.34 | 22.17 | 24.32 |
| **Gelatin peptone_7_R1_P4** | 16.50 | 19.05 | 18.51 | 17.64 | 24.04 | 21.92 | 21.19 | 16.81 | 17.99 | 19.18 | 24.50 | 22.48 | 24.68 |
| **Gelatin peptone_7_R2_P4** | 16.67 | 19.17 | 17.93 | 17.79 | 24.21 | 22.57 | 21.38 | 17.28 | 18.02 | 19.37 | 24.64 | 22.56 | 24.76 |
| **Gelatin peptone_7_R3_P4** | 16.19 | 19.07 | 17.89 | 17.43 | 24.04 | 22.16 | 21.24 | 17.40 | 18.07 | 19.31 | 24.72 | 22.38 | 24.17 |

| SampleID | 18S | Actb | B2m | Gapdh | Hmbs | Hprt | Nono | Ppia | Rplp0 | Ywhaz | Tbp | Pparg | Fasn |
|---|---|---|---|---|---|---|---|---|---|---|---|---|---|
| **Gelatin peptone_7_R4_P5** | 16.67 | 19.50 | 18.44 | 18.02 | 24.52 | 22.68 | 21.47 | 17.62 | 18.33 | 19.60 | 24.73 | 22.74 | 23.46 |
| **Gelatin peptone_7_R5_P5** | 16.07 | 18.87 | 18.26 | 18.00 | 24.20 | 22.40 | 21.36 | 17.48 | 18.34 | 19.55 | 24.72 | 22.69 | 24.36 |
| **Gelatin peptone_7_R6_P5** | 16.56 | 19.09 | 18.00 | 18.02 | 24.49 | 22.60 | 21.67 | 17.38 | 18.21 | 19.51 | 24.71 | 22.56 | 25.93 |
| **Gelatin peptone_14_R1_P4** | 15.90 | 16.20 | 18.55 | 17.32 | 25.12 | 23.27 | 23.06 | 18.88 | 18.43 | 20.57 | 24.85 | 22.72 | 24.51 |
| **Gelatin peptone_14_R2_P4** | 16.30 | 17.93 | 18.45 | 17.38 | 25.18 | 23.22 | 22.68 | 18.81 | 18.29 | 20.28 | 25.07 | 22.66 | 25.28 |
| **Gelatin peptone_14_R3_P4** | 16.10 | 17.40 | 18.83 | 17.26 | 25.12 | 23.51 | 22.47 | 18.91 | 18.48 | 20.50 | 25.07 | 22.40 | 25.07 |
| **Gelatin peptone_14_R4_P5** | 16.40 | 17.52 | 19.10 | 18.07 | 25.36 | 23.43 | 22.63 | 19.02 | 18.88 | 20.59 | 25.44 | 23.22 | 24.51 |
| **Gelatin peptone_14_R5_P5** | 16.40 | 17.52 | 18.63 | 17.18 | 25.12 | 23.32 | 22.53 | 18.85 | 18.36 | 20.38 | 24.98 | 23.00 | 24.31 |
| **Gelatin peptone_14_R6_P5** | 16.50 | 17.53 | 18.77 | 17.67 | 25.30 | 23.48 | 22.38 | 18.99 | 18.56 | 20.69 | 23.67 | 23.14 | 25.00 |

Sample IDs encode the culture substrate (TCP, collagen, gelatin peptone, or Matrigel), culture duration (7 or 14 days), biological replicate number (R1–R6), and cell passage number (P4 or P5). For example, TCP_7_R1_P4 indicates tissue culture plastic (TCP), 7 days of culture, biological replicate 1, and passage 4.
